# Single-cell profiling of immune cells after neoadjuvant pembrolizumab and chemotherapy in IIIA non-small cell lung cancer (NSCLC)

**DOI:** 10.1038/s41419-022-05057-4

**Published:** 2022-07-13

**Authors:** Zhenzhen Hui, Jiali Zhang, Yulin Ren, Xiaoling Li, Cihui Yan, Wenwen Yu, Tao Wang, Shanshan Xiao, Yulong Chen, Ran Zhang, Feng Wei, Jian You, Xiubao Ren

**Affiliations:** 1 Tianjin Medical University Cancer Institute and Hospital, National Clinical Research Center for Cancer, Tianjin, 300060 China; 2 Tianjin’s Clinical Research Center for Cancer, Tianjin, 300060 China; 3 Key Laboratory of Cancer Immunology and Biotherapy, Tianjin, 300060 China; 4 Department of Biotherapy, Tianjin Medical University Cancer Institute and Hospital, Tianjin, 300060 China; 5 Department of Immunology, Tianjin Medical University Cancer Institute and Hospital, Tianjin, 300060 China; 6 International Personalized Cancer Center, Tianjin Cancer Hospital Airport Hospital, Tianjin, 300308 China; 7 Department of R&D, Hangzhou Repugene Technology Co., Ltd., Hangzhou, 311100 China; 8 Department of Lung Cancer, Tianjin Lung Cancer Center, Tianjin Medical University Cancer Institute and Hospital, Tianjin, 300060 China; 9 Department of Thoracic Oncology Surgery, Tianjin Cancer Hospital Airport Hospital, Tianjin, 300308 China

**Keywords:** Non-small-cell lung cancer, Cancer immunotherapy, Cancer microenvironment

## Abstract

The combination of immune checkpoint inhibitors (ICIs) with chemotherapy (chemoimmunotherapy) in the neoadjuvant setting have achieved favorable clinical benefits in non-small cell lung cancer (NSCLC), but the mechanism of clinical responses remain unclear. We provide a rich resource of 186,477 individual immune cells from 48 samples of four treatment-naive and eight neoadjuvant chemoimmunotherapy treated IIIA NSCLC patients (responders versus non-responders) by single-cell RNA-seq and TCR-seq. We observed the synergistic increase of B cells and CD4^+^ T cells were associated with a positive therapeutic response of neoadjuvant chemoimmunotherapy. B cell IgG subclasses IgG1 and IgG3 played a critical role in anti-tumor immune response in tumor lesions, and this process was driven by increased IL-21 secreted by infiltrated T follicular helper (Tfh) cells after neoadjuvant chemoimmunotherapy. Furthermore, we uncovered several critical events for positive clinical outcomes, including the diminished activated TNFRSF4^+^ regulatory T cells (Tregs), increased LAMP3^+^ dendritic cells (DCs), and the expansion of intratumoral CD4^+^ T clones and peripheral C3-Cytotoxic CD8^+^ T clones. A validation cohort of 26 treatment-naive and 30 neoadjuvant chemoimmunotherapy treated IIIA/ IIIB NSCLC patients verified these findings. In total, our comprehensive study of the single-cell profile of immune cells provides insights into mechanisms underlying anti-PD-1-based therapies and identified potential predictive factors and therapeutic targets for improving the efficiency of neoadjuvant chemoimmunotherapy in NSCLC.

## Introduction

Lung cancer continues to be the leading cause of cancer death worldwide. Approximately 20% of patients with non-small cell lung cancer (NSCLC) are diagnosed with stage IIIA (N2) disease with poor outcomes [1], and neoadjuvant chemotherapy has been recommended to shrink the tumors [2]. However, neoadjuvant chemotherapy often causes immune cell toxicity, escape, and resistance that is most frequently associated with tumor recurrence. Perioperative platinum-based chemotherapy is associated with a survival rate that is only 5.4% higher than that with surgery alone [3]. Even after curative resection, ~60% of patients with stage IIIA disease still develop recurrence after 3 years [4].

Recently, neoadjuvant immunotherapy for resectable NSCLC has emerged as a powerful treatment option for advanced NSCLC. Several studies have demonstrated the effectiveness and feasibility of neoadjuvant therapy with immune checkpoint inhibitors (ICIs) in potentially resectable melanoma, glioblastoma, and NSCLC [5–7]. PD-1 blockage given prior to surgical removal of NSCLC is safe and induced a major pathological response (MPR) in 45% of resected tumors, according to the first neoadjuvant immunotherapy trial for patients with early-stage lung cancer [5]. Preliminary research also found that higher levels of PD-L1 expression and T cell subsets regulation in non-responders to platinum-based neoadjuvant chemotherapy, suggesting the benefit for combined immunotherapy and chemotherapy (refer to chemoimmunotherapy) prior to surgical resection of locally advanced NSCLC [8]. The synergistic effects of combining immunotherapy with chemotherapy have been demonstrated in several clinical trials, thus providing the proof-of-concept that neoadjuvant chemoimmunotherapy plays a significant role in the multimodality therapy of early-stage resectable NSCLC. Two major open-label, multicenter, single-arm phase 2 trials have shown beneficial clinical responses of neoadjuvant chemoimmunotherapy. One is NADIM, conducted at 18 hospitals in Spain demonstrated that could increase the proportion of patients with resectable NSCLC who achieve a complete pathological response (pCR), and that this approach would increase the number of patients that can ultimately be cured [9]. The other one performed at three US hospitals showed that Atezolizumab plus carboplatin and nab-paclitaxel could be a potential neoadjuvant regimen for resectable NSCLC, with 57% of patients achieving major pathological response (MPR) and no compromised surgical resection [10]. In a single-center study of 35 patients with resectable stage IIIA/IIIB NSCLC who received neoadjuvant pembrolizumab and chemotherapy, 51.43% patients achieved pCR and 74.29% patients achieved MPR, and PD-L1 expression (TPS with 50% threshold) in biopsy tumor tissue prior to treatment was not associated with neoadjuvant pathological response [11]. Despite the unprecedented clinical success of neoadjuvant ICIs and chemotherapy, the underlying molecular mechanism has not been fully elucidated and the differences in treatment response have been connected to heterogeneity in the immune cell composition of individual tumors.

To identify factors associated with the success or failure of neoadjuvant ICIs and chemotherapy, we performed single-cell transcriptome and TCR sequencing on CD45^+^ immune cells from 12 surgical resected IIIA NSCLC patients. We examined the tumor immune transcriptomic profiles and their association with the clinical response to neoadjuvant chemoimmunotherapy at single-cell solution, including the composition of immune cells, the diversity, the expansion, and dynamics of intratumoral and peripheral T-cell clonotypes. We uncovered several critical events for positive clinical outcomes, including the co-increase of CD4^+^ T cells and B cells, B-cell isotype switching, the diminishment of TNFRSF4^+^ regulatory T cells (Tregs), increased LAMP3^+^ dendritic cells (DCs), the expansion of intratumoral CD4^+^ T clones and peripheral CD8^+^ T clones, etc. In total, our comprehensive study of single-cell profiling of immune cells reveals previously unrecognized predictors, mechanisms and targets for enhancing the clinical responses of chemoimmunotherapy and helps select the most suitable NSCLC patients who are more likely to benefit from this neoadjuvant chemoimmunotherapy.

## Results

### Single-cell profiling of immune cells of lung tumors and their association with response to neoadjuvant chemoimmunotherapy

**Fig. 1:**
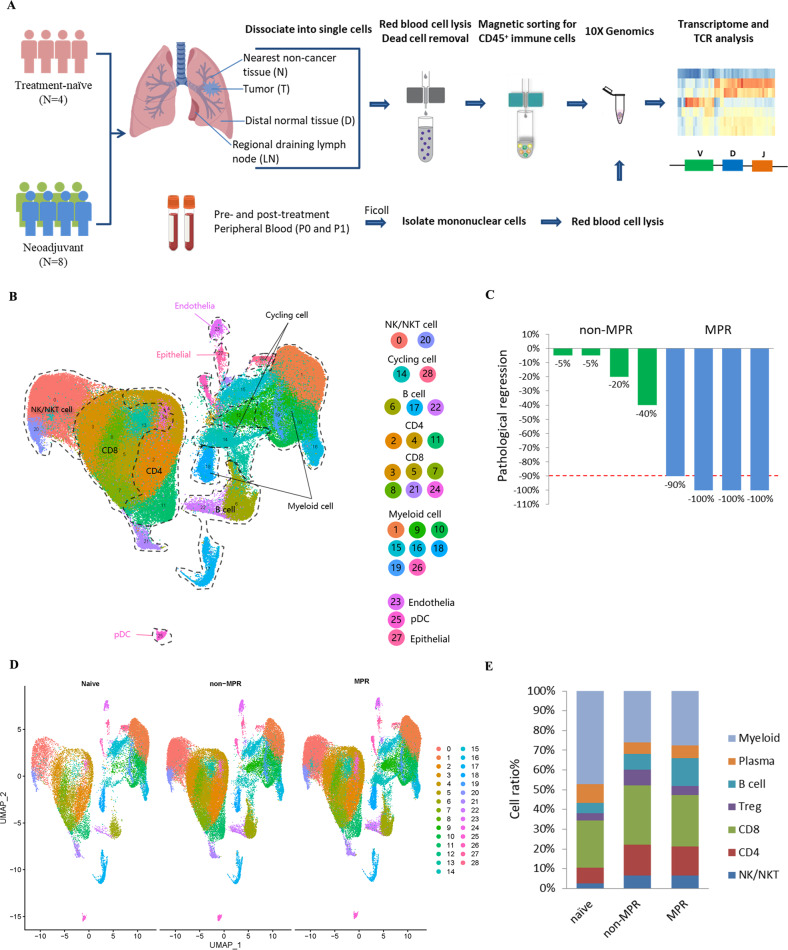
Clustering and dominant immune cells associated with neoadjuvant response. **A** Flow chart of the overall study design (Figure created with biorender.com.). CD45^+^ immune cells derived from tumor tissues (T) and five immune-relevant sites (nearest non-cancer tissues (N), regional draining lymph nodes (LN), distal normal tissues (D), peripheral blood mononuclear cells before and after neoadjuvant therapy (hereafter we called P0 and P1)) were applied to single-cell transcriptome/TCR sequencing. **B** The UMAP projection of 186,477 cells from 12 patients, showing the formation of 29 main clusters, including two for NK/NKT cells, three for B cells and plasma cells, three for CD4^+^ T cells, six for CD8^+^ T cells, eight for myeloid clusters and two cycling cells. Each dot corresponds to one single cell, colored according to the cell cluster. **C** Neoadjuvant patient categories according to their pathologic response, with four patients in MPR and four patients in non-MPR groups. **D** UMAP displaying of the immune cell composition in treatment-naive, non-MPR and MPR tumor tissues. **E** Composites of major immune cell proportions in treatment-naive, non-MPR, and MPR tumor tissues.

To analyze immune cells associated with the clinical efficacy of neoadjuvant chemoimmunotherapy, we provided single-cell RNA and paired T-cell receptor (TCR) sequencing profiles on CD45^+^ immune cells isolated from lung cancer tumor tissues (T) and matched five immune-relevant sites (nearest non-cancer tissues (N), regional draining lymph nodes (LN), distal normal tissues (D), peripheral blood mononuclear cells before and after neoadjuvant therapy (hereafter we called P0 and P1)) of 12 IIIA (N2) NSCLC patients, including four treatment-naive and eight neoadjuvant pembrolizumab and chemotherapy patients (Fig. 1A, Supplementary Table S1). After quality-control filtering, we acquired single-cell transcriptomes on a total of 186,477 immune cells, with 39,792 from tumor tissues, 34,042 from nearest non-cancer tissues, 18,577 from regional draining LNs, 27,786 from distal normal tissues, 19,509 from P0 and 46,741 from P1 samples (Supplementary Fig. S1A). To explore the cellular composition in an unbiased manner, we applied principal component analysis on variably expressed genes across all cells to generate uniform manifold approximation and projection (UMAP) and identified 29 main clusters, including two clusters enriched for NK/NKT cells (C0-NK/NKT-GNLY, C20-NK-XCL1), three clusters for CD4^+^ T cells (C2-CD4-IL7R, C4-CD4-TCF7, C11-Treg-FoxP3), six clusters for CD8^+^ T cells (C3-CD8-KLRG1, C5-CD8-IL7R, C7-CD8-PDCD1, C8-CD8-GZMK, C21-CD8-MKI67, C24-CD8-TCF7), three B and plasma cell clusters (C6-B cells-CD79A, C17-Plasma cells-IGHG1, C22-B cells-MS4A1), nine myeloid clusters (C1-TAM-CD81, C9-MDSC-like macrophage-S100A8/A9, C10-Neutrophil-CXCL8, C15-Macrophage-CCL18, C16-DC-CD1C, C18-Mast cells-TPSAB1, C19-Monocyte-FCGR3A, C25-pDC-IL3RA, C26-Megakaryocyte-PPBP), as well as C23-Endothelial cells-VWF, C27-Epithelial cells-KRT17 and cycling cell clusters such as C14-MALAT1 and C28-STMN1 (Fig. 1B, Supplementary Fig. S1B, Supplementary Table S2).

To address which immune cell type associates with a therapeutic response of neoadjuvant pembrolizumab and chemotherapy, we analyzed the tumor-infiltrating immune cell composites in treatment-naive, neoadjuvant MPR, and non-MPR patients. MPR is an endpoint defined as ≤10% of viable tumor cells in a surgically resected specimen, which has been adopted in post neoadjuvant therapy and may predict disease-free survival (DFS) and overall survival (OS) in various clinical trials [12]. Therefore, we introduced MPR as the early surrogate endpoint in our analysis and divided eight neoadjuvant pembrolizumab and chemotherapy patients into non-MPR and MPR groups, according to the pathologic response evaluated by two senior clinical pathologists, with four patients in MPR and four patients in non-MPR groups (Fig. 1C). We identified significant differences in lymphocyte and myeloid cell composition in neoadjuvant tumor lesions, with lymphocytes constituted 47.55%, 65.81%, and 64.82% in each treatment-naive, neoadjuvant non-MPR and MPR tumor lesions respectively, while myeloid cells constituted 42.54%, 23.25%, and 24.61% respectively. Compared with treatment-naive tumor lesions, more intra-tumoral NK/NKT and CD4^+^ T cells were found in neoadjuvant non-MPR and MPR tumor lesions (NK/NKT: 2.36% versus 5.71% versus 5.72%; CD4^+^ T cells: 7.23% versus 14.05% versus 13.24%). We also noted that B cells were more abundant in MPR tumor lesions than treatment-naive and non-MPR (12.69% versus 4.65% versus 7.00%), while Tregs were of relative higher number in non-MPR tumor lesions than those in treatment-naive and MPR tumor lesions (7.10% versus 3.38% versus 4.14%; Fig. 1D, E), suggesting differential anti-tumor response may exist in different clinical outcome of neoadjuvant pembrolizumab and chemotherapy.

### B cells class switched to IgG1 and IgG3 isotype and their association with neoadjuvant chemoimmunotherapy response

**Fig. 2:**
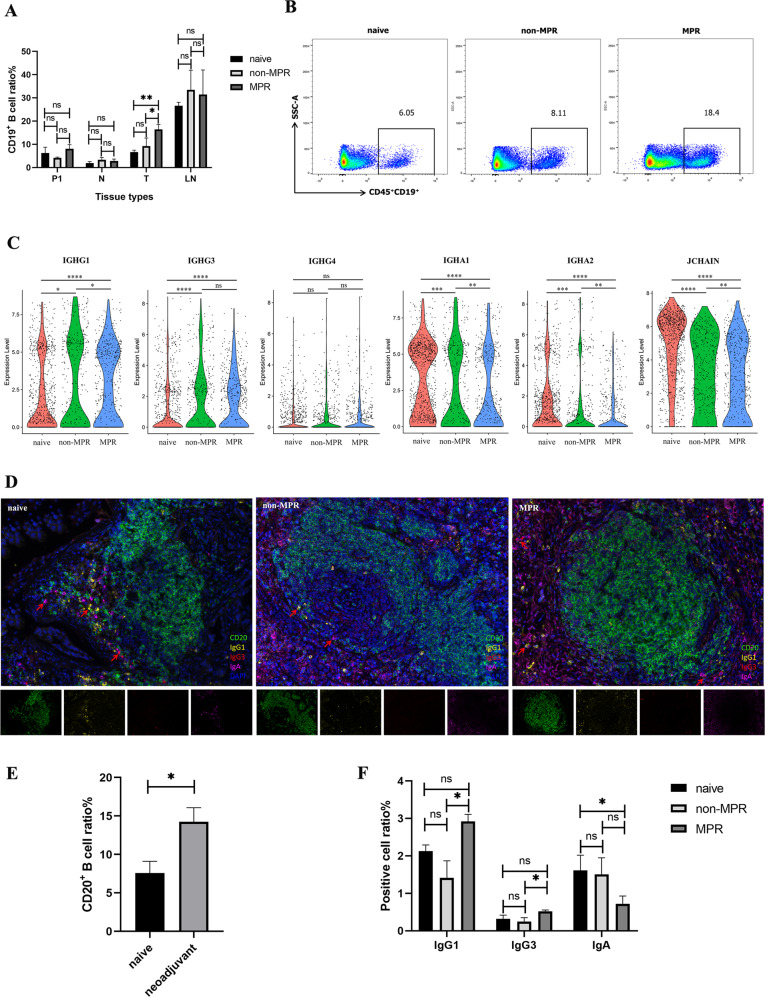
B cells and their class switching to IgG-mediated neoadjuvant response. **A**, **B** Comparison of CD19^+^ B cells in tumor, nearest non-cancer tissues (N), LNs, and P1 in different treatment groups by Flow cytometry. Data presented as mean ± SEM. Data were summarized from *n* = 8 treatment-naive, *n* = 5 non-MPR and *n* = 12 MPR tumor and relevant tissue samples. *P* values were determined by ordinary one-way ANOVA. *p < 0.05, **p < 0.01, ns: not significant. **C** The violin plot tested with Wilcoxon showed increased IGHG1 and IGHG3, decreased IGHA expression by C17-plasma cells in neoadjuvant responder tumor lesions. *p < 0.05, **p < 0.01, ***p < 0.001, ****p < 0.0001, ns: not significant. **D** Multiplex fluorescent IHC (mIHC) staining of surgical tumor tissues showed the density and spatial distribution of B cells isotypes in single-cell sequencing cohort. **E**–**F** Comparison of CD20^+^ B cells and immunoglobulin (Ig) isotypes proportions in different therapy groups. Data presented as mean ± SEM. Data were summarized from *n* = 6 treatment-naive, *n* = 6 non-MPR and *n* = 6 MPR tumor samples. *P* values were determined by the Kruskal–Wallis test, two-tailed. *p < 0.05, ns: not significant.

Given B cells were remarkably enriched in neoadjuvant MPR tumor lesions in our single-cell RNA profiles, we speculated that B cells may be a key determinant of therapeutic response to neoadjuvant pembrolizumab and chemotherapy. To confirm this hypothesis, we performed flow cytometry analysis and identified a striking abundance of CD19^+^ B cells in tumor lesions of MPR patients compared with treatment-naive and non-MPR patients (Fig. 2A, B, Supplementary Fig. S2A, B), and this proportion of CD19^+^ B cells were positively correlated with therapy response, while there was no difference in nearest non-cancer tissues (N), LNs and P1.

To gain deeper insight into the function of B cells, we compared the differentially expressed genes (DEGs) of C17-plasma cells (Supplementary Fig. S2, C) in treatment-naive, neoadjuvant non-MPR, and MPR tumor lesions. We found IGHA1, IGHA2, and JCHAIN were significantly decreased in neoadjuvant MPR tumor lesions than in non-MPR tumor lesions, while IGHG1 and IGHG3 were significantly upregulated in MPR tumor lesions (Fig. 2C), indicating the B cells class switched to two dominant antibody isotype IgG1 and IgG3 during neoadjuvant chemoimmunotherapy. To confirm this, we next assessed tumor samples histologically to gain insights into the density and distribution of B cells isotypes by multiplex fluorescent IHC staining (mIHC), and demonstrated that neoadjuvant chemoimmunotherapy promoted more intratumoral CD20^+^ B cells infiltration (mean 7.59% versus 14.25%, Fig. 2D, E). And consistent with our scRNA findings, IgG1, and IgG3 positive cells showed an increasing trend in MPR tumor lesions (mean IgG1: 2.13% versus 1.41% versus 2.92%; mean IgG3: 0.32% versus 0.25% versus 0.52%), while IgA positive cells showed a decreasing trend in MPR tumor lesions (mean 1.62% versus 1.51% versus 0.72%; Fig. 2D, F). These data indicated the potential for B cell class switching and antibody responses (increased IgG1 and IgG3, diminished IgA) induced by neoadjuvant chemoimmunotherapy to promote favorable anti-tumor immune response.

### Interleukin (IL)-21 induced B cell isotype switching (IgG1 and IgG3) and its association with neoadjuvant chemoimmunotherapy response

**Fig. 3:**
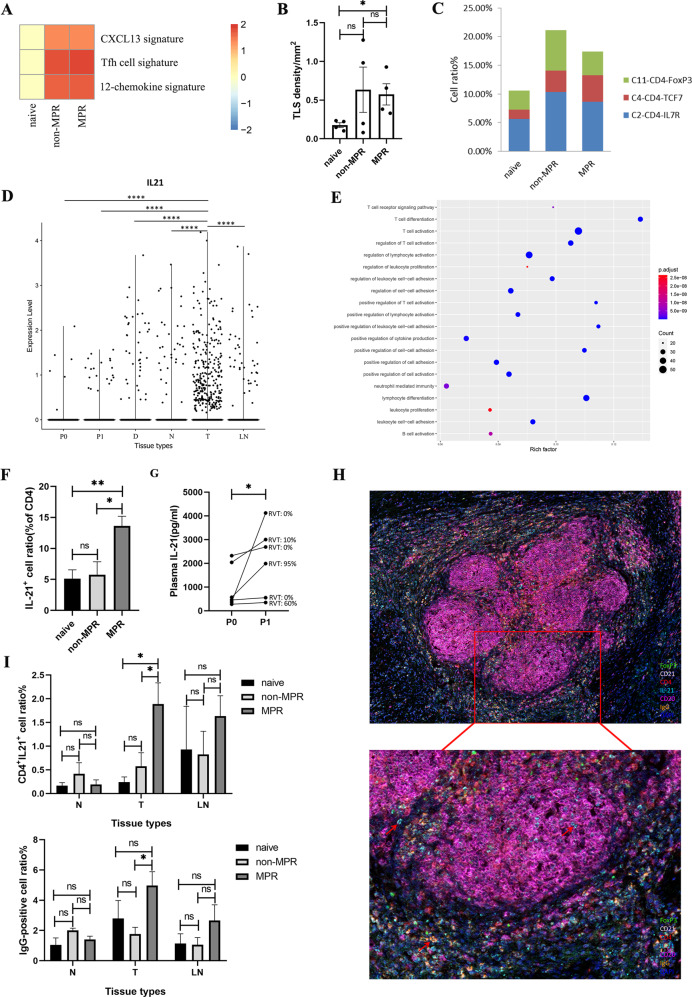
IL-21 secreted by Tfh drives B cell class switching to IgG in tumor lesion. **A** The heatmap showing GSEA enrichment of the CXCL13, Tfh cell, and 12-chemokine signatures in treatment-naive, non-MPR, and MPR tumor tissues. *P*adj > 0.05 in the enrichment pathway was adjusted to 0 to draw the heat map. **B** The higher TLS density in neoadjuvant tumor lesions verified by mIHC staining in a single-cell sequencing cohort. **C** Higher intratumoral C2-CD4-IL7R and C4-CD4-TCF7 cluster proportions in neoadjuvant tumor lesions in scRNA profiles. **D** IL21 expression in different tissue types tested with Wilcoxon in scRNA profiles showing that IL21 was most prevalent in tumor tissues. ****p < 0.0001. **E** Go biological function analysis of the IL21 expression cells by clusterprofiler in scRNA profiles. **F** Flow cytometry of IL-21 in CD4^+^ T cells in tumor tissues. Data presented as mean ± SEM. Data were summarized from *n* = 8 treatment-naive, *n* = 5 non-MPR and *n* = 15 MPR tumor samples. *P* values were determined by ordinary one-way ANOVA. *p < 0.05, **p < 0.01, ns: not significant. **G** Comparison of paired plasma IL-21 before and after neoadjuvant chemoimmunotherapy. *P* values were determined by Wilcoxon matched-pairs signed-rank test, two-tailed. *p < 0.05. **H**, **I** MIHC staining showing CD4^+^IL21^+^ and IgG-positive cells were much abundant in MPR tumor tissues (image under 10× and 20×). Data presented as mean ± SEM. Data were summarized from *n* = 6 treatment-naive, *n* = 9 non-MPR and *n* = 18 MPR tumor samples. *P* values were determined by ordinary one-way ANOVA. *p < 0.05, ns: not significant.

To explore the transcriptome characteristics in our single-cell profiles, we applied gene set enrichment analysis (GSEA) to detect signatures in tumor tissues of different therapy groups, including Tfh cell signature (a distinct subset of CD4^+^ helper T cells that regulate the development of antigen-specific B cell immunity), CXCL13 signature (dominant chemokine for B cell and Tfh cells chemotaxis and germinal center formation) and the 12-chemokine signature (for detection of tertiary lymphoid structures (TLSs) [13]. We found that Tfh cell signature was much abundant in neoadjuvant tumor lesions, indicating their potentially important role in the anti-tumor immune response of neoadjuvant chemoimmunotherapy. The CXCL13 signature and the 12-chemokine signature were also significantly enriched in neoadjuvant tumor lesions, indicating increased lymphocytes infiltration and TLS formation after neoadjuvant chemoimmunotherapy (Fig. 3A). To validate this notion, we performed mIHC on surgical tumor tissues, and observed higher TLS density (number/mm^2^) in neoadjuvant tumor lesions versus treatment-naive tumor lesions (Fig. 3B). Furthermore, we checked the composition of intratumoral CD4^+^ T cells in our scRNA profile and also confirmed a higher proportion of C2-CD4-IL7R and C4-CD4-TCF7 clusters in neoadjuvant chemoimmunotherapy treated tumor lesions (C2-CD4-IL7R: 5.60% versus 10.35% versus 8.61%; C4-CD4-TCF7: 1.63% versus 3.70% versus 4.63%; Fig. 3C), indicating that the B-cell enrichment accompanied with an elevated level of CD4^+^ T cells in neoadjuvant tumor lesions. Moreover, C4-CD4-TCF7 cluster overexpressed transcription factors TCF7 and LEF1, which orchestrate Tfh cell differentiation by regulating differentiation circuits upstream of the transcriptional repressor Bcl6 [14].

Several studies have demonstrated the critical role of B cells and TLSs in therapeutic response to ICIs in patients with melanoma, sarcoma, and RCC [15–17]. But the function of B cells in this process is not completely understood. Previous studies have demonstrated that IL-21 plays a crucial role in T cell-dependent B cell differentiation into plasma cells and memory cells, stimulation of IgG production, and induction of apoptotic signaling in naive B cells and is mainly produced by Tfh cells, NK cells, and T helper (Th)17 [18–21]. To check if IL-21 is involved in neoadjuvant chemoimmunotherapy induced B cell class switching, we analyzed the characteristics of these IL21-producing cells in our scRNA data and found that they were most prevalent in tumor tissues (Fig. 3D). Furthermore, these cells possessed Tfh-like features with high expression of CXCL13, IL21, PDCD1, TOX2, ICOS, BATF, MAF, SAP (SH2D1A), TIGIT, CD40LG as well as the B cell differentiation factor Blimp-1 (PRDM1) (Supplementary File S1), suggesting Tfh cells are the main immune cells to secret IL-21 [22]. High expression of CXCL13 in these IL-21 producing cells induced B-cell chemotaxis toward lymphatic follicles and facilitated their interactions with Tfh cells, as well as IL-21 primarily produced by Tfh cells, which are necessary for B-cell activation and differentiation. To assess the biological function of these IL21-producing cells, we performed GO functional enrichment analysis of the above DEGs, and found they were mainly concentrated in T cell activation, lymphocyte differentiation, positive regulation of cell–cell adhesion, positive regulation of cytokines production, and B cell activation (Fig. 3E). To further confirm the potential role of IL-21 in mediating chemoimmunotherapy response, we used flow cytometry analysis and demonstrated that CD4^+^IL21^+^ cells were significantly higher in MPR tumor tissues than those in treatment-naive and non-MPR tumor tissues (Fig. 3F). Collectively, neoadjuvant pembrolizumab and chemotherapy increased Tfh cell infiltration and IL-21 secretion within the tumor tissues to promote anti-tumor response.

Plasma IL-21 level was also elevated after neoadjuvant chemoimmunotherapy in our single-cell study cohort, and higher IL-21 concentration was consistent with less residual viable tumor cells (RVT) (Fig. 3G). Next, to investigate the expression and distribution characteristics of IL-21, IgG, and TLS in the tumor microenvironment, we performed mIHC on 30 surgical tumor tissues following neoadjuvant chemoimmunotherapy and identified more CD4^+^IL21^+^ and IgG-positive cells in MPR tumor tissues (Fig. 3H, I, Supplementary Fig. S3), while there was no difference in nearest non-cancer tissues (N), and LNs. Taken all together, our analysis suggested that B cells class switched to IgG subclass mediated favorable anti-tumor immune response during neoadjuvant chemoimmunotherapy, and that increased IL-21 secreted by CD4^+^ T cells was the driving force in tumor lesion, although further functional studies are needed to verify this.

### Reinvigoration and clonal expansion of CD8^+^ T cells induced by neoadjuvant chemoimmunotherapy

**Fig. 4:**
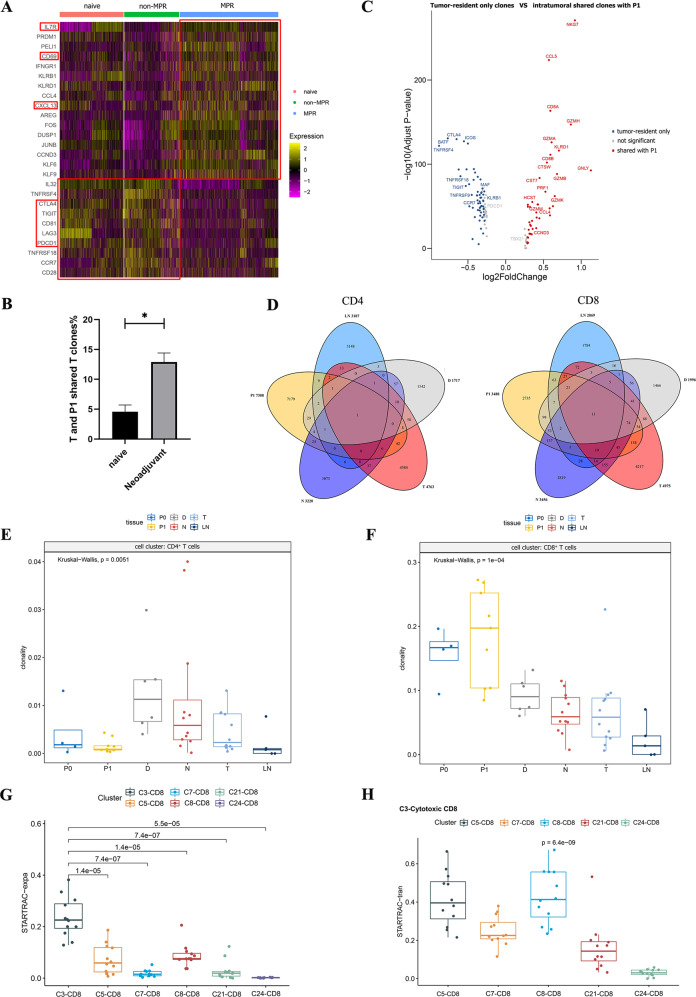
Reinvigoration and clonal expansion of CD8^+^ T cells and their association with neoadjuvant response. **A** Heatmap showing the DEGs of CD8^+^ T cells in treatment-naive, non-MPR, and MPR tumor tissues. The exhausted state of CD8^+^ T cells was greatly improved in MPR tumor lesions. B. Comparison of shared T clone between Tumor and P1 in treatment-naive and neoadjuvant tumor lesions. There are more shared T clones between tumors and P1 in neoadjuvant tumor lesions. Data presented as mean ± SEM. *P* value was determined by the Mann–Whitney test, two-tailed. *p < 0.05. **C** The volcano map shows the comparison of DEGs between tumor-resident only T clones and shared T clones with P1 in tumor lesions, the shared T clones were more cytotoxic, while tumor-resident only T clones were more exhausted. **D** Venn diagram showing the shared clonotype between different tissues, there are more shared CD8^+^ T clones than CD4^+^ T clones between tissues. **E**, **F** Comparison of the clonality of CD4^+^ and CD8^+^ T cells in different tissue types. The CD8^+^ T clones were widely expanded in various tissues, and were more obvious in P1 than P0. *P* values were determined by the Kruskal–Wallis test. **G** STARTRAC-expa index revealed the C3-cytotoxic CD8^+^ T cluster with the highest degree of clonal expansion compared to other CD8^+^ T clusters. **H** STARTRAC-tran analysis indicated that the C3-cytotoxic CD8^+^ T cluster was highly associated with both C5-CD8-IL7R and C8-CD8-GZMK clusters. *P* values were determined by the Wilcoxon test, two-tailed.

To understand the functional status of T cells in different treatment groups, we compared the DEGs of CD4^+^ and CD8^+^ T clusters in treatment-naive, non-MPR, and MPR tumor lesions. The data revealed that the exhaustion status of CD8^+^ T cells was significantly improved in MPR tumor lesions, with reduced expression of exhausted markers, such as PDCD1, CTLA4, LAG3, and TIGIT. In addition, memory and survival marker IL7R, tissue-resident marker CD69, and lymphocyte chemokine CXCL13 were also upregulated in MPR (Fig. 4A). High expression of CXCL13 in CD8^+^ T cells may promote lymphocyte chemotaxis across high endothelial venule into tumor lesions to support TLS formation and was associated with ICIs therapy response [23, 24]. The relatively high diversity of T cell clonotypes in non-MPR and MPR tumor lesions hinted that new T clones with new TCR repertoires may influx into tumor lesions after neoadjuvant therapy (Supplementary Fig. S4A, B). Since pre-existing tumor-specific T cells have limited reinvigoration capacity and exhausted CD8^+^ T cells could not acquire a memory/effector T-cell phenotype due to their epigenetic stability [25, 26], clonal replenishment of T cells from the periphery may contribute to the improvement of the CD8^+^ T cell exhausted state. To verify this idea, we compared shared T clones between tumors and P1 and found that the shared T clones with P1 were significantly increased in neoadjuvant tumor lesions (Fig. 4B). Then, we compared the DEGs between tumor and P1 shared T clones and tumor-resident only T clones in tumor lesions and identified an abundant expression of CD8A and CD8B in shared T clones, and the shared CD8^+^ T clones upregulated cytotoxic genes such as NKG7, GZMH, GZMA, GZMK, KLRD1, GNLY, GZMB, PRF1, CTSW, CST7, GZMK, and GZMM, while tumor-resident only CD8^+^ T clones were highly expressed CTLA4, ICOS, TIGIT, CD161 (KLRB1), CCR7 and PDCD1, consistent with the notion that intrinsic tumor-resident T cell repertoire may likely to be exhausted and dysfunctional with low tumor reactivity (Fig. 4C) [25]. To address the potential migration trajectories of T cells, we analyzed the clonotypes shared among five related samples including tumor tissues, nearest non-cancer tissues, LN, distal normal tissues and P1, and identified more shared CD8^+^ T clones between different tissues than CD4^+^ T clones (Fig. 4D). Overall, we noted more CD8^+^ T clones were shared between tumor lesions with P1 (7.64% of the total intratumoral CD8^+^ T clones), then nearest non-cancer tissues (7.14% of the total intratumoral CD8^+^ T clones), whereas tumor-infiltrating CD4^+^ T cells shared more clones with distal normal tissues (only 1.26% of the total intratumoral CD4^+^ T clones). The high degree of inter-tissue TCR distribution implied that these T cells with the same ancestors may migrate between different tissues, and the migration of CD8^+^ T cells between different tissues was more active than in CD4^+^ T cells. Thus, neoadjuvant pembrolizumab and chemotherapy improve the exhausted and dysfunctional state of intratumoral CD8^+^ T cells in responders, possibly by promoting clonal replenishment of T cell clones from peripheral blood.

Next, we explored the clonal expansion of CD4^+^ and CD8^+^ T cells in different tissue types separately and found obvious discrepancies (Fig. 4E, F; Supplementary Fig. S4C, D). The CD4^+^ T clones were slightly expanded in tumor tissues (T), nearest non-cancer tissues (N), and distal normal tissues (D), reflecting the local activation and expansion of intra-tissues CD4^+^ T clones happened during neoadjuvant pembrolizumab and chemotherapy. Whereas CD8^+^ T clones were highly expanded in various tissues and were more obvious in P1 than P0 and other tissues, suggesting CD8^+^ T clones have been activated and expanded in the periphery following neoadjuvant pembrolizumab and chemotherapy. These data indicated that neoadjuvant pembrolizumab and chemotherapy evoke the expansion of intra-tissue CD4^+^ T clones and peripheral CD8^+^ T clones to promote anti-tumor immune response. To track clonal cell fates during chemoimmunotherapy, we matched clonotypes between P0 and P1 based on TCR sequence, and demonstrated that the majority of matched T clones between P0 and P1 were CD8^+^ T cells, and the matched CD8^+^ T clones were more expanded in P1 than P0 (Supplementary Fig. S4E, F). The dynamic of these matched clonotypes supported our notion that chemoimmunotherapy could reinvigorate the clonal expansion of peripheral CD8^+^ T cells. Furthermore, the STARTRAC-expa index revealed the C3-cytotoxic CD8^+^ T cluster with the highest degree of clonal expansion compared to other CD8^+^ T clusters (Fig. 4G). STARTRAC-tran analysis indicated that C3-cytotoxic CD8^+^ T cluster was highly associated with both C5-CD8-IL7R and C8-CD8-GZMK clusters, indicating the state transition between different CD8^+^ T clusters (Fig. 4H). P0 and P1 pairwise analysis before and after neoadjuvant chemotherapy revealed an increase in NK/NKT and CD8^+^ T cells and a decrease in CD4^+^ T cells, especially a significant increase in C3-cytotoxic CD8^+^ T cells after neoadjuvant chemoimmunotherapy (Supplementary Fig. S4G, H). Collectively, these results demonstrated that the reinvigoration and clonal expansion of peripheral C3-cytotoxic CD8^+^ T clones is a key factor affecting the neoadjuvant chemoimmunotherapy response.

### Diminished TNFRSF4^+^ Tregs in tumor lesion and their association with the positive therapy response

**Fig. 5:**
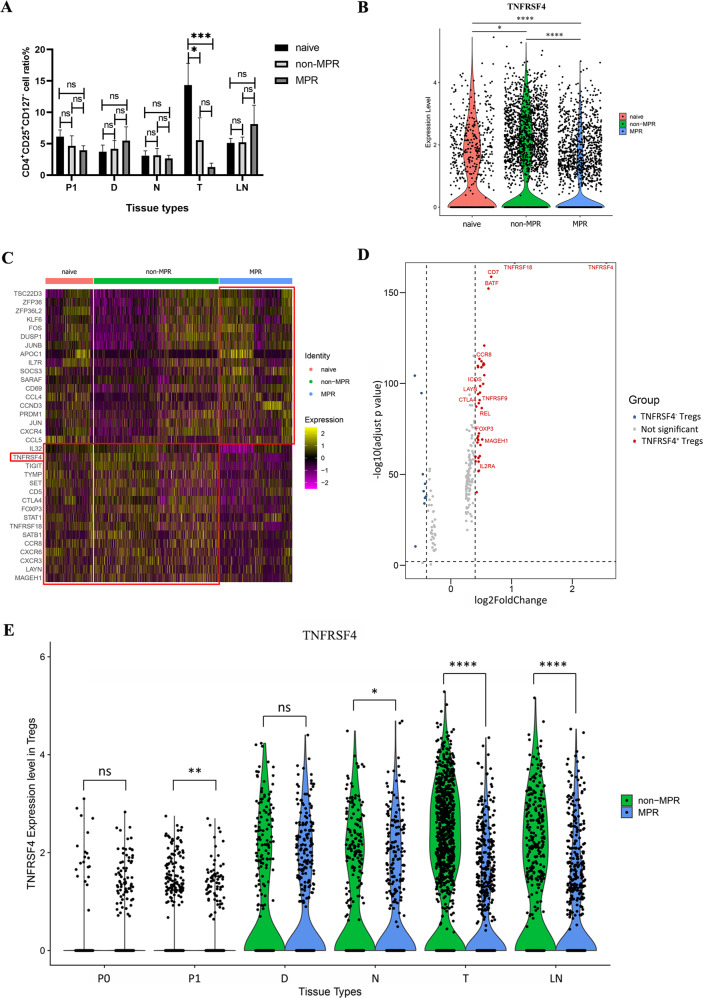
Diminished TNFRSF4^+^ Tregs in tumor lesion predict positive therapy response. **A** Comparison of CD4^+^CD25^+^CD127^−^ Treg cells in different tissue types of treatment-naive, non-MPR, and MPR groups by flow cytometry. The ratio of CD4^+^CD25^+^CD127^−^ cells was striking reduced in MPR tumor lesions. Data presented as mean ± SEM. Data were summarized from *n* = 8 treatment-naive, *n* = 5 non-MPR and *n* = 15 MPR tumor samples. *P* values were determined by ordinary one-way ANOVA. *p < 0.05, ***p < 0.001, ns: not significant. **B** The violin of TNFRSF4 expression in C11-Treg cluster of treatment-naive, non-MPR, and MPR patients in scRNA profiles. *p < 0.05, ****p < 0.0001. **C** Heatmap showing the DEGs of C11-Tregs-FoxP3 in treatment-naive, non-MPR, and MPR tumor tissues. **D** The volcano map showing the comparison of DEGs in TNFRSF4^+^ and TNFRSF4^−^ Treg cells, the TNFRSF4^+^ Tregs were more activated and immunosuppressive. **E** Comparison of TNFRSF4 expression in C11-Tregs-FoxP3 of different tissues showing that the expression of TNFRSF4 was significantly decreased in P1, the nearest non-tumor tissue (N), tumor tissue (T), and LNs in MPR than non-MPR patients, and was especially lower in MPR tumor tissues compared with non-MPR tumor lesions. *P* values were determined by the Wilcoxon test. *p < 0.05, **p < 0.01, ****p < 0.0001, ns: not significant.

Regulatory T cells (Tregs) play a critical role in mediating immune tolerance and tumor escape [27]. One interesting phenomenon we noticed is that C11-Tregs-FoxP3 cluster were more abundant in non-MPR tumor lesions, indicating their aggregation suppressed the immune response induced by neoadjuvant chemoimmunotherapy in non-responder patients (Fig. 1E). Flow cytometry analysis also confirmed a decreased proportion of CD4^+^CD25^+^CD127^−^ Treg cells in MPR tumor lesions, consistent with the negative association of Tregs to the clinical response to neoadjuvant chemoimmunotherapy (Fig. 5A). Moreover, the costimulatory molecule TNFRSF4 (OX40), top of the marker genes of C11-Tregs-FoxP3 cluster in our scRNA profiles, was found in significantly lower level in MPR patients than in treatment-naive and non-MPR patients (Fig. 5B).

To further evaluate the immunosuppression of Tregs in different treatment groups, we generated the DEGs of C11-Tregs-FoxP3 in treatment-naive, non-MPR, and MPR patients. We found that besides TNFRSF4, Treg functional signature genes such as FoxP3, CTLA4, TIGIT, TNFRSF18 (GIRT), CCR8, LAYN, and MAGEH1 were also overexpressed in C11-Tregs-FoxP3 of treatment-naive and non-MPR tumor lesions, but not for MPR tumor lesions (Fig. 5C). To explore the functional differences between TNFRSF4^+^ and TNFRSF4^−^ Treg cells, we compared TNFRSF4^+^ and TNFRSF4^−^ Treg cells in the C11-Tregs-FoxP3 cluster. The volcanic map of DEGs showed that TNFRSF4^+^ Tregs were more activated and immunosuppressive with high expression of Treg functional signatures such as GITR (TNFRSF18), CCR8, LAYN, TNFRSF9, CTLA4, REL, FoxP3, MAGEH1, and IL2RA (Fig. 5D), which was correlated with poor prognosis in NSCLC, colorectal cancer (CRC) and breast cancer (BC) patients [28–32]. To validate their association with therapeutic response, we made a comparison of TNFRSF4 expression among C11-Tregs-FoxP3 of different tissue types of neoadjuvant non-MPR and MPR patients, and demonstrated that the expression of TNFRSF4 was significantly lower in P1, nearest non-tumor tissue (N), tumor tissue (T) and LNs in MPR than non-MPR patients, which was strikingly lower in MPR tumor tissues compared with non-MPR tumor lesions (Fig. 5E). Therefore, neoadjuvant chemoimmunotherapy could eliminate the activated immunosuppressive TNFRSF4^+^ Tregs, and the diminished levels of TNFRSF4^+^ Tregs may be used as a positive predictor of neoadjuvant chemoimmunotherapy response.

### LAMP3^+^ DCs participated in the recruitment and regulation of lymphocytes during neoadjuvant chemoimmunotherapy

**Fig. 6:**
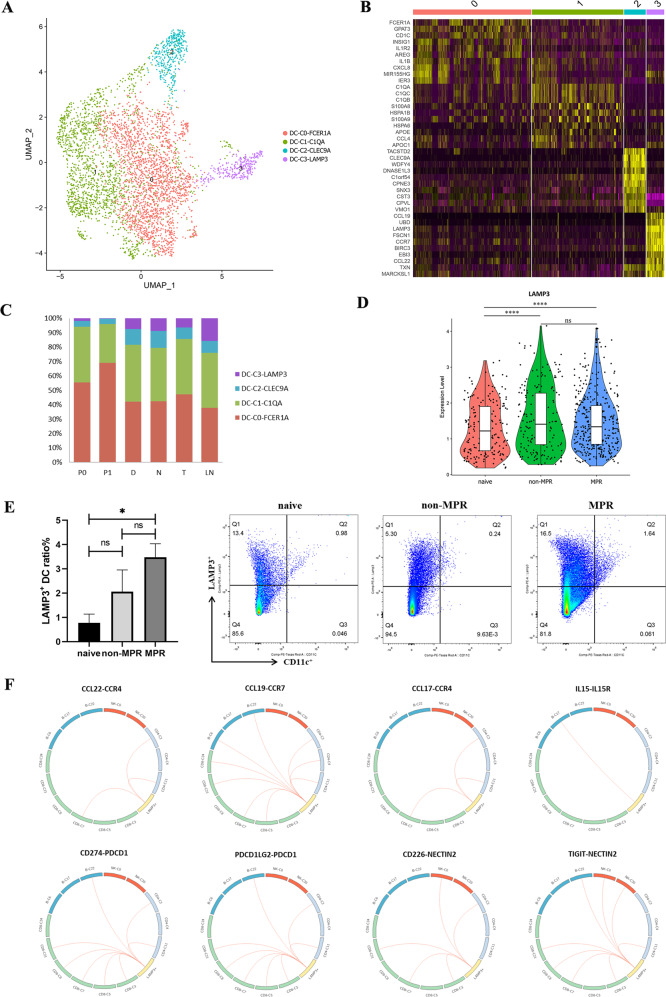
Characteristics and L-R interactions of DCs and Lymphocytes and their association with clinical outcome. **A** UMAP plot showing the clusters of DC subsets. Each dot represents a single cell. Color represents cluster. **B** Heatmap showing the DEGs of DC subsets. The maturation markers LAMP3, chemokine, and chemokine receptor CCL19, CCR7, and CCL22 were highly expressed in DC-C3-LAMP3. **C** Tissue distribution analysis of the four DC clusters, DC-C3-LAMP3 was mainly distributed in tumors, LNs, and nearest or distal normal tissues, and was rarely seen in peripheral blood. **D** The violin plot tested with Wilcoxon showing LAMP3 expression level in treatment-naive, non-MPR and MPR patients. ****p < 0.0001, ns: not significant. **E** LAMP3^+^ DC ratio in tumor tissues detected by flow cytometry. The proportion of LAMP3^+^ DC cells was significantly higher in neoadjuvant MPR tumor lesions. Data presented as mean ± SEM. Data were summarized from *n* = 4 treatment-naive, *n* = 6 non-MPR and *n* = 6 MPR tumor samples. *P* values were determined by the Kruskal–Wallis test. *p < 0.05, ns: not significant. **F** L-R based interaction of LAMP3^+^ DCs and lymphocytes predicted by CellPhone DB.

Dendritic cells (DCs) are central regulators of adaptive immune responses and are necessary for T/B cell-mediated cancer immunity [33]. We detected 3671 dendritic cells in our scRNA analysis, and four distinct subtypes were revealed by sub-clustering analysis (Fig. 6A), including DC-C0-FCER1A, DC-C1-C1QA, DC-C2-CLEC9A, and DC-C3-LAMP3. We next attempted to identify marker genes for these four clusters. DC-C0-FCER1A expressed a high level of FCER1A, CD1C, and CLEC4A, corresponding to cDC2, whereas DC-C3-CLEC9A expressed a high level of TACSTD2, CLEC9A, and CADM1, representing cDC1. Cells in DC-C3-LAMP3 have highly expressed the maturation markers LAMP3, CD83, and CD80; the migration marker CCR7; and the lymphocyte recirculation chemokine CCL19, CCL22, and CCL17, suggesting that DC-C3-LAMP3 was a group of mature DCs originate from other clusters and possess the potential of migrating from tumors to LNs (Fig. 6B). We observed a higher proportion of cDC2s than cDC1s in multiple tissues. Particularly, DC-C3-LAMP3 was mainly distributed in tumors, LNs, and nearest or distal normal tissues, but was rarely seen in P0 and P1 (Fig. 6C). Then, in order to explore the role of LAMP3^+^DCs in neoadjuvant chemoimmunotherapy, we compared the expression of LAMP3 in different treatment groups and found the expression of LAMP3 was significantly higher in neoadjuvant patients than in treatment-naive patients (Fig. 6D). Flow cytometry analysis also demonstrated that the ratio of LAMP3^+^DCs in neoadjuvant MPR tumor lesions was significantly higher than that in treatment-naive tumor tissues, and displayed an increasing trend in MPR than non-MPR tumor tissues (Fig. 6E). These results indicate that LAMP3^+^ DCs are involved in and positively modulate the therapeutic response to neoadjuvant pembrolizumab and chemotherapy.

To further investigate the interactions between LAMP3^+^ DCs and lymphocytes, we utilized a set of immune-related ligand-receptor (L-R) pair analyses to gain insights into the regulatory relationships among cell clusters. DC-C3-LAMP3 was predicted to interact with C2-CD4-IL7R, C11-CD4-Treg and C7-CD8-PDCD1 clusters via the CCL22/CCR4 and CCL17/CCR4 axes, and interact with the CD4^+^ T cells, CD8^+^ T cells and B cells through CCL19/CCR7 axes, indicating a potential role of LAMP3^+^ DCs for lymphocytes recruitment during neoadjuvant chemoimmunotherapy (Fig. 6F). In addition, a pluripotent cytokine-interleukin (IL)-15, overexpressed by LAMP3^+^ DCs, has been reported as an immunotherapeutic agent for the treatment of cancer [34]. IL-15 plays multiple roles in activating T cells, B cells, NK cells, but not for Tregs [35]. LAMP3^+^ DCs were predicted to interact with C17-plasma cells through IL15/IL15R pathway to support B cells proliferation and differentiation in our L-R analysis (Fig. 6F). Furthermore, we found that LAMP3^+^ DCs exhibit the regulation activity towards B cells, Tregs, CD4^+^ and CD8^+^ T lymphocytes through PD1/PD-L1 and PD1/PD-L2 signaling pathways, as well as NK/NKT cells via CD226/NECTIN2 and TIGIT/NECTIN2 pathways, and macrophage and DCs cells via CD80 interacted with PD-L1 to disrupt PD-L1/PD-1 binding to promote anti-tumor T cell response [36] (Fig. 6F, Supplementary Figs. S5, S6). Altogether, our data suggested that LAMP3^+^ DCs are involved in the recruitment and regulation of multiple lymphocyte subsets via L-R interactions, and are more likely associated with the abundant intratumoral lymphocyte infiltration and the clinical response to neoadjuvant pembrolizumab and chemotherapy.

### Increased B cell subclasses (IgG1 and IgG3) and CD4^+^ T cells in TLSs and their association with positive clinical effects in the validation cohort

**Fig. 7:**
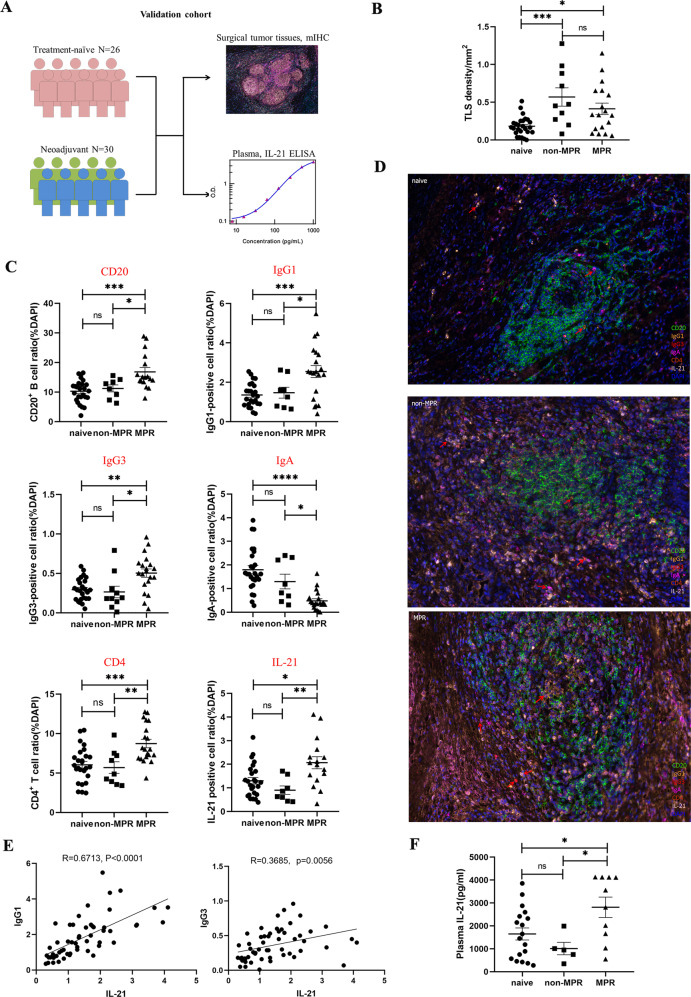
Increased B cell subclass (IgG1 and IgG3) and CD4^+^ T cells in TLSs and their association with positive clinical effect in the validation cohort. **A** Validation cohort: Surgical resected tumor tissues from 26 treatment-naive IIIA/IIIB NSCLC and 30 resectable IIIA/IIIB NSCLC who have received two cycles of neoadjuvant pembrolizumab and chemotherapy before surgery. **B** Neoadjuvant chemoimmunotherapy promoted more TLSs formation in non-MPR and MPR tumor lesions in our validation cohort. Data presented as mean ± SEM. Data were summarized from *n* = 26 treatment-naive, *n* = 10 non-MPR and *n* = 20 MPR tumor samples. *P* values were determined by ordinary one-way ANOVA. *p < 0.05, ***p < 0.001, ns: not significant. **C**, **D** MIHC staining of B cell isotypes, CD4 and IL-21 showing that CD20, IgG1, IgG3, CD4, and IL-21 were significantly higher in MPR tumor lesions, whereas IgA was much lower in MPR tumor lesions. Data presented as mean ± SEM. Data were summarized from *n* = 26 treatment-naive, *n* = 10 non-MPR and *n* = 20 MPR tumor samples. *P* values were determined by ordinary one-way ANOVA. *p < 0.05, **p < 0.01, ***p < 0.001, ****p < 0.0001, ns: not significant. **E** Pearson correlation coefficient analysis found that IgG1 and IgG3 were positively correlated with IL-21 within tumor lesions. **F** Plasma IL-21 was significantly higher in MPR patients than that in treatment-naive and non-MPR patients in our validation cohort. Data presented as mean ± SEM. Data were summarized from *n* = 18 treatment-naive, *n* = 5 non-MPR and *n* = 10 MPR tumor samples. *P* values were determined by ordinary one-way ANOVA. *p < 0.05, ns: not significant.

To validate factors identified in our study that affect the therapeutic response to neoadjuvant chemoimmunotherapy, we performed mIHC staining on different treatment groups with antibodies CD20, IgG1, IgG3, IgA, CD4, IL-21, including 26 treatment-naive IIIA/IIIB NSCLC tumor tissues and 30 surgical tumor specimens from patients diagnosed with IIIA/IIIB NSCLC and received neoadjuvant pembrolizumab and chemotherapy before surgery (Validation cohort, Fig. 7A). The clinicopathological characteristics of the validation cohort were well balanced with those of the patients selected for single-cell sequencing analysis (Supplementary Table S3). The mIHC results showed that neoadjuvant chemotherapy promoted more TLSs formation in non-MPR and MPR tumor lesions compared to treatment-naive patients in our validation cohort (Fig. 7B). Specifically, compared with treatment-naive and non-MPR patients, neoadjuvant MPR patients had more CD20^+^ B cell infiltration and a significantly higher proportion of B-cell subclasses IgG1 and IgG3 positive cells, and the ratio of IgA positive cells was significantly lower than that in treatment-naive and non-MPR tumor tissues. The proportion of CD4 and IL-21 positive cells in MPR tumor tissues were also significantly higher than that in treatment-naive and non-MPR tumor tissues (Fig. 7C, D, Supplementary Fig. S7). Furthermore, the ratio of IgG1 and IgG3 positive cells was positively correlated with the number of IL-21 positive cells within tumor lesions (Fig. 7E). These data indicated the relative abundance of distinct antibody subclasses in the tumor microenvironment is an important predictor of chemoimmunotherapy response. Next, we compared these markers in different therapeutic response groups of adenocarcinoma (AD) and squamous cell carcinoma (SCC) patients, respectively. We found that higher levels of CD4, CD20, IL-21, IgG1, and IgG3 and lower levels of IgA in MPR tumor lesions of AD and SCC patients after neoadjuvant chemoimmunotherapy, although statistical significance was not reached in AD due to the small number of cases, suggesting that similar antitumor immune responses occur in the local tumor microenvironment of AD and SCC patients during neoadjuvant chemoimmunotherapy (Supplementary Table S4). Collectively, we confirmed that the increased number of B cell subclasses IgG1 and IgG3 and CD4^+^ T cells, and increased level of IL-21 in responder tumor lesions, played critical roles in shaping anti-tumor immune responses and the positive therapeutic response of neoadjuvant chemoimmunotherapy in clinic.

To verify whether the level of IL-21 in peripheral blood plasma can reflect the changes in the local tumor microenvironment and whether it is related to the clinical response of neoadjuvant pembrolizumab and chemotherapy, we checked the concentration of plasma IL-21 by ELISA collected from 17 treatment-naive patients and 13 neoadjuvant pembrolizumab and chemotherapy patients in our validation cohort. Our results demonstrated that plasma IL-21 was significantly higher in MPR patients than that in treatment-naive and non-MPR patients (Fig. 7F). Taken all together, our result provided the possibility of plasma IL-21 as a novel potential factor for predicting patients who are most likely to benefit from chemoimmunotherapy and monitoring therapeutic response in dynamic.

## Discussion

**Fig. 8:**
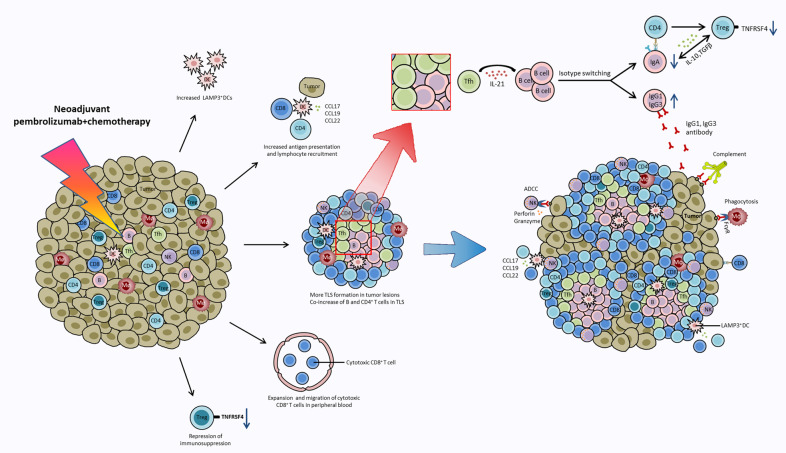
Summary of key anti-tumor events occurring during neoadjuvant chemoimmunotherapy. Neoadjuvant chemoimmunotherapy promotes LAMP3^+^ DCs aggregation in tumor lesions, and the increased LAMP3^+^ DCs acquire tumor antigen, migrate and interact with lymphocytes, and participate in the activation, recruitment, and regulation of T/B lymphocytes through L-R interactions. Anti-PD-1 therapy reinvigorates the expansion of intratumoral CD4^+^ T cells and peripheral cytotoxic CD8^+^ T clones and promoted CD8^+^ T cells migration to tumor tissues to exert an anti-tumor effect. Neoadjuvant chemoimmunotherapy promotes more TLSs formation in NSCLC tumor tissues. In tumor-associated TLSs, IL-21 secreted by Tfh cells promotes B cells class switching to IgG1 and IgG3 but not IgA to mediate anti-tumor response. IgG1 and IgG3 antibodies bind to Fcγ receptor (FcγR) and trigger ADCC and antibody-mediated phagocytosis and mediate complement-based cytotoxicity. In addition, the decrease of immunosuppressive IgA^+^plasma cells and TNFRSF4^+^ Tregs lead to the repression of immunosuppression during chemoimmunotherapy.

To our knowledge, our study is the first published immune landscape to explore the molecular and cellular mechanism of neoadjuvant pembrolizumab and chemotherapy at single-cell resolution in patients with resectable NSCLC. Based on our findings, we proposed a hypothesis that several critical anti-tumor events happened during neoadjuvant chemoimmunotherapy (Fig. 8). Neoadjuvant chemoimmunotherapy promoted more TLSs formation in surgical NSCLC tumor tissues. The synergistic increase of B cells and CD4^+^ T cells in TLSs are associated with a positive therapeutic response. This is in line with several studies showing that the presence of TLSs, B, and Tfh cells are correlated with prolonged survival and favorable therapeutic responses [20, 37–39]. A central finding in our study is that B cell subclasses IgG1 and IgG3 play a critical role in anti-tumor response to neoadjuvant chemoimmunotherapy, and IL-21 (mainly secreted by Tfh cells) is the driving force to induce B cell isotype switching to IgG1 and IgG3, not IgA in tumor lesion. We also found that IL21R was overexpressed in plasma cells (C22), T cells (C2, C3, C5, C7, C8, C11, C12, C21), and NK cells (C20), suggesting that IL-21 secreting cells may interact with these cells via paracrine action. In addition, the increase in TLS in tumor tissue after neoadjuvant chemoimmunotherapy may provide an ideal site for tumor-specific T-B cell interactions and collaboration. Published studies have confirmed that IL-21 can induce class switching of naive B cells to IgG [40, 41], and IL-21 stimulation up-regulated STAT3 phosphorylation, increased Blimp-1 expression in B cells, and promoted plasma cell differentiation. The dominant IgG1 and IgG3 antibodies can bind to the Fcγ receptor (FcγR) and trigger ADCC and antibody-mediated phagocytosis and mediate complement-based cytotoxicity to kill tumor cells [42]. In contrary, the reduced IgA isotype has an immunosuppressive effect and promotes the transition and expansion of Treg cells, which reciprocally produce transforming growth factor-β (TGF-β) mediating B cells class switching to IgA [43]. Consistent with our findings, Bassez and colleagues [44] performed single-cell sequencing and proteomic analysis of tumor tissue from breast cancer patients before and after anti-PD-1 therapy and demonstrated that CD4^+^ T cells characterized by expression of T helper 1 (IFNG) and Tfh (BCL6, CXCR5) markers clonally expanded upon anti-PD1 treatment, suggesting that Tfh plays a critical role in mediating the therapeutic response to anti-PD-1 based therapy. Cui et al. [22] also demonstrated that B cells promote the differentiation of tumor-specific Tfh cells in a neoantigen-dependent manner, which in turn enhances the effector function of CD8^+^ T cells by producing IL-21 and drives anti-tumor immunity in a murine model of lung adenocarcinoma. Fusing engineered IL-21 with existing immunotherapies can elicit robust anti-tumor immune responses [45, 46]. Vaccines, agonist antibodies, or cytokines targeting B cell-Tfh-IL21 can provide therapeutic benefits [47–49]. Taken together, these data suggested that therapeutic designs leveraging the B cell-Tfh-IL21 axis may be one of the strategies to boost anti-tumor immune response and further research is needed to translate these findings into the clinic.

Yost et al. [25] have demonstrated that pre-existing tumors specific T cells may have limited reinvigoration capacity, and that the T cell response to ICIs derives from a distinct repertoire of T cell clones that may have just recently entered the tumor from periphery. Although PD-1 blockage has been reported to reinvigorate peripheral T cell clones that are dynamic exchanged with the tumor by a TCR Vβ complementarity-determining region 3 (CDR3) sequencing study [50], they did not really distinguish whether it was primarily CD4^+^ or CD8^+^ T clones that were relevant to the pathologic responses of PD-1 blockage. In our scRNA profiles, we analyzed the expansion, diversity and trafficking of CD4^+^ and CD8^+^ T cells separately based on our scTCR profile, demonstrating the reinvigoration of CD8^+^ T clones following chemoimmunotherapy, in particular, the expansion of C3-Cytotoxic CD8^+^ T cells in P1 were associated with improved immunological responses. We also found that neoadjuvant chemoimmunotherapy promoted more shared T-cell clones between tumor and P1, and the exhaustion status of intratumoral CD8^+^ T cells in MPR tumor lesions was significantly improved. Based on these findings, we hypothesized that tumor-resident T clones may dynamically exchange with peripheral blood, anti-PD-1 therapy reinvigorates neoantigen-specific CD8^+^ T clones in peripheral blood, and peripherally expanded CD8^+^ T cells migrated to tumor lesions to produce the anti-tumor response. Our finding was also supported by Han and his colleagues’ study which sequenced the (CDR3) of PD-1^+^CD8^+^ TCR Vβ in peripheral blood before and after ICIs therapy [51], suggesting that the diversity and clonality of CD8^+^ T cells in peripheral blood may serve as noninvasive predictors of patient response and survival outcomes in NSCLC. Thus, the dynamic monitoring of CD8^+^ T cells and their TCR repertoire may be an early biological correlation of anti-tumor T cell recognition.

Tregs are master immunoregulatory cells in tumor microenvironments [52]. Our study demonstrated that one Treg subset TNFRSF4^+^ Tregs were inversely correlated with neoadjuvant chemoimmunotherapy response. TNFRSF4^+^ Tregs featured with high expression of Treg functional signature genes and exhibited the strongest immune-suppressive function compared to TNFRSF4^−^ Tregs. Consistently, it has been reported that Treg cells with the expression of TNFRSF4 were more immunosuppressive and facilitated tumor immune evasion, and promote tumor development in nasopharyngeal carcinoma, cutaneous squamous cell carcinoma and chronic myeloid leukemia [53–55]. Anti-TNFRSF4 antibody augmented antitumor immunity in animal models with several types of cancers, and the anti-tumor effects were mainly dependent on the reduction of Treg cells in tumors. In mice, the combination of anti-PD-1 inhibitory and anti-TNFRSF4 antibodies reduces the proportion of Tregs and exhausted T cells in pancreatic tumors and increases the numbers of memory CD4^+^ and CD8^+^ T cells, eradicating all detectable tumors, which can be used in the development of immune-based combination therapy [56]. Thus, the diminished activated TNFRSF4^+^ Tregs in tumor lesions are associated with the positive clinical outcome and could be used as potential predictors and therapeutic targets for neoadjuvant chemoimmunotherapy.

Garris et al. demonstrated that the successful anti-tumor immune response of PD-1 inhibitors depends on the interaction between T cells and DCs [57]. LAMP3^+^ DCs are a subset of mature DCs derived from cDC1 and cDC2, which can migrate from tumors to LNs and initiate antigen-specific immune responses [58]. Mature LAMP3^+^ DCs were also reported to exist in multiple tumor types and were associated with increasing tumor-infiltrating lymphocytes in the local tumor lesion [59]. A pan-cancer single-cell transcriptome analysis of myeloid cells confirmed that although the proportion of cDC2 was much higher in tumor tissues, cDC1-derived LAMP3^+^ cDCs were more abundant than cDC2-derived ones. LAMP3^+^ DCs from different origins maintain specific transcriptome characteristics and might exhibit different functions [60]. Our study demonstrated a remarkable increase of intratumoral LAMP3^+^ DCs aggregation in tumor lesions in neoadjuvant MPR tumor lesions, suggesting LAMP3^+^ DCs may contribute to the anti-tumor immune response of neoadjuvant chemoimmunotherapy. We also explored the possible mechanisms by which LAMP3^+^ DCs recruit and regulate lymphocytes through multi-pair L-R interaction analysis, illustrating a potential role for LAMP3^+^ DCs in immunotherapy. However, due to the complex co-expression of activated and inhibitory molecules, further studies are needed to validate these findings.

Taken together, our extensive immune single-cell RNA-seq and TCR-seq landscape provide novel insights into the cellular mechanisms underlying the synergistic interaction in clinical responses to neoadjuvant chemoimmunotherapy. Of note, our study showed that enrichment of CD4^+^ T and B cells correlated with favorable clinical outcomes in NSCLC, and that IL-21 is critical for tumor control and B cells class switching to anti-tumor IgG1 and IgG3 isotypes. Based on this finding, IL-21 in combination with ICIs may improve antitumor immunity, and clinical trials are needed to verify this. Moreover, the detection of IL-21 in peripheral blood plasma is non-invasive, convenient and feasible, and can be used as a potential predictor of clinical response to chemoimmunotherapy and even for monitoring the efficacy of chemoimmunotherapy in advanced NSCLC. Lastly, our study identified TNFRSF4 as a potential target to reduce the function of Tregs and improve anti-tumor immunity against NSCLC.

Limitations of this study include: (1) the relatively small number of patients enrolled and incomplete samples for some patients; (2) not reached longer follow-up for DFS and OS benefit; and (3) scRNA profiles of AD and SCC could not be analyzed separately due to the small number of patients enrolled. Further large-scale studies are needed to unravel the difference between AD and SCC subtypes, as well as to validate potential predictors and strategies to improve the clinical outcome of combined immunotherapy and chemotherapy.

## Materials and methods

### Patient cohorts

Twelve patients who were diagnosed with stage IIIA NSCLC were enrolled in the single-cell sequencing analysis, including four treatment-naive and eight neoadjuvant pembrolizumab and chemotherapy patients, with five adenocarcinoma and seven squamous cell carcinoma patients. The basic clinicopathological characteristics of these patients are summarized in Supplementary Table S1. Surgical tumor tissues and paired adjacent non-cancer tissues and LNs from an additional 26 treatment-naive IIIA/IIIB NSCLC and 30 neoadjuvant pembrolizumab and chemotherapy IIIA/IIIB NSCLC patients were used to perform mIHC to validate the findings identified in the scRNA-seq profiles (Validation cohorts). The clinicopathological characteristics of enrolled patients were comparable in single-cell sequencing and validation cohorts (Supplementary Table S3). This study was approved by the Ethics Committee of Tianjin Medical University Cancer Institutes and Hospital (NO.: bc2020060), and conformed to the Declaration of Helsinki and Good Clinical Practice guidelines. Informed consent was obtained from all patients.

For the patients included in the neoadjuvant pembrolizumab and chemotherapy group, none of the patients had autoimmune disease, interstitial lung disease or prior cancer history. No ongoing glucocorticoid or immunosuppressant usage. No previous treatment of checkpoint inhibitors or other drugs that target T-cell co-stimulation or immune checkpoint pathways. Patients with an acute or chronic hepatitis virus infection or active tuberculosis were also excluded. The included patients received neoadjuvant treatment with intravenous pembrolizumab (at a dose of 2 mg per kilogram of body weight) on day 1, paclitaxel 175 mg/m^2^ for squamous cell carcinoma, or pemetrexed 500 mg/m^2^ for adenocarcinoma plus carboplatin (area under curve 5; 5 mg/mL per min) on day 1, 21 days each cycle, for two cycles before surgical resection, and then followed by two cycles after surgical resection.

### Clinical sample collection and preparation

The fresh NSCLC tumors and matched adjacent non-cancer tissues, regional draining lymph nodes, and distal normal lung tissues, as well as pre- and post-treatment peripheral blood, were obtained for subsequent CD45^+^ immune cells isolation. The adjacent non-cancer tissues were defined as 1–2 cm from the matched tumor tissues. Distal normal lung tissues were sampled 10–15 cm from the tumor margin of surgically resected specimens. Post-treatment venous blood was taken prior to surgery and other tissues were collected in 1640 medium (Gibco, Cat# 11875085) within 5 min after bulk tumor resection and placed on ice.

Peripheral blood mononuclear cells (PBMCs) were isolated by ficoll density separation using HISTOPAQUE-1077 (Sigma-Aldrich, Cat# 10771) solution according to the manufacturer's instructions. Specifically, 10 ml pre- and post-treatment were collected in EDTA anticoagulant tubes, and centrifuged at 2000 rpm/min for 10 min. The upper plasma was partitioned and stored at −80 °C, and lower cell precipitates were diluted with DPBS (no calcium, no magnesium, Gibco, Cat# 14190-136) and subsequently layered onto HISTOPAQUE-1077. After centrifugation, immune cells remaining at the DPBS-HISTOPAQUE-1077 interface were carefully transferred to a new tube and washed twice with 1× DPBS. Then, these immune cells were diluted with 10 volumes of 1× red blood cell lysis solution (Miltenyi, Cat# 130-094-183), vortex for 5 s, and incubate for 10 min at room temperature. Centrifuged at 300 × *g* for 10 min, aspirated supernatant completely and the cell pellet were resuspended in sorting buffer (DPBS supplemented with 0.5% BSA) and proceeded to 10× Genomics.

Fresh tumor, adjacent non-cancer tissues, regional draining lymph nodes, and distal normal lung tissues were cut into ~1–3 mm³ pieces and in the RPMI-1640 medium (Gibco, Cat# 11875085) with 10% fetal bovine serum (FBS; Gibco, Cat# 12484028). The pieces were transferred to the gentle MACS C Tubes (Miltenyi Biotec, Cat# 130-096-334), with 5 mL of digestive enzyme included in Tumor Dissociation Kit (Miltenyi Biotec, Cat# 130-095-929). Then the tissues were made into single-cell suspension using the gentleMACS Dissociator (Miltenyi Biotec, Cat# 130096427) for 60 min on a rotor at 37 °C according to the manufacturer’s instructions. After filtered by 70 μm cell-strainer (BD Falcon, Cat# 352350) in the RPMI-1640 medium, the suspended cells were centrifuged at 300 × *g* for 10 min. After removing the supernatant, the pelleted cells were suspended in 1× red blood cell lysis solution (Miltenyi, Cat# 130-094-183), votex for 5 s and incubated at room temperature for 2 min to lyse red blood cells. Dead cell and cellular debris were magnetically removed using Dead Cell Removal Kit (Miltenyi Biotec, Cat# 130-090-101). The affluent live cell fraction was centrifuged at 300 × *g* for 10 min. The cell pellets were re-suspended in sorting buffer (DPBS supplemented with 0.5% BSA) after washing twice with DPBS. CD45^+^ immune cells were obtained by positive magnetic cell sorting using CD45^+^ cell isolation beads (CD45 MicroBeads, human, Miltenyi Biotec, Cat# 130-045-801) according to the manufacturer’s instructions. CD45^+^ immune cells were centrifuged at 300 × *g* for 10 min, resuspended, and stained with 0.4% Trypan blue (Gibco, Cat# 15250061) to check the viability, adjusted cell concentration to 700–1200 cells/μL for 10x Genomics single-cell sequencing.

### Library construction for single-cell RNA sequencing and TCR profiling

Cell suspensions (700–1200 living cells/μL determined by trypan blue staining) were loaded on a 10x Genomics ChromiumTM Single Cell Controller Instrument (10x Genomics, Pleasanton, CA, USA) to generate single-cell gel beads in emulsions (GEMs) by using Chromium Single Cell 5′ Library & Gel Bead Kit v1.0 (10x Genomics, Cat# 1000006) and Single Cell A Chip Kit (10x Genomics, Cat# 1000152). Captured cells were lysed and the released RNA was barcoded through reverse transcription in individual GEM. Barcoded cDNAs were pooled and cleanup by using DynaBeads MyOne Silane Beads (Thermo Fisher, Cat# 37002D), and then amplified and cleanup for further next-generation library construction. Single-cell RNA-seq libraries were prepared using Chromium Single Cell 5′ Library & Gel Bead Kit v1.0 (10x Genomics, Cat# 1000006) and i7 Multiplex Kit (10x Genomics, Cat# 120262), following the manufacturer’s instructions.

TCR libraries were prepared using Chromium Single Cell V(D)J Enrichment Kit, Human T Cell (10x Genomics, Cat# 1000005), Chromium Single Cell 5′ Library Construction Kit (10x Genomics, Cat# 1000020), and i7 Multiplex Kit (10x Genomics, Cat# 120262), following the manufacturer’s instructions. Sequencing was performed on an Illumina HiSeq X Ten (Illumina, San Diego, CA, USA) with pair end 150 bp (PE150).

### scRNA analysing

Cell Ranger 3.1.0 was used to align and quantify the generated scRNA-seq data against the GRCh38 human reference genome. After generating the gene expression matrix, we filtered the cell-identifying barcodes to avoid dead cells and other artifacts with Seurat 3.2.1. Briefly, we filter out cells with <200 or >6000 genes detected per cell, or with <1000 UMI, or with more than 10% mitochondrial genes. We used the FindIntegrationAnchors (MNN algorithm) function in the Seurat package to correct the batching effect between samples [61]. The final expression table contained 186,477 cells.

### Unsupervised clustering, DEGs, and pathway analysis

We used the Seurat package for unsupervised clustering of cells based on gene expression profiles and passed it to UMAP for cluster visualization [62]. The top 50 principal components in principal component analysis (PCA) were adopted to construct the Shared nearest-neighbor graph (SNN graph), which was completed by the ‘FindNeighbors’ function in Seurat package. Louvain algorithm [63] was used to cluster the data set, which was completed by the ‘FindClusters’ in the Seurat package. We tested the resolution of the ‘FindClusters’ from 0.2–1.0, and found that 0.8 was the most appropriate resolution. Cells were displayed on a two-dimensional UMAP plane, and clusters were identified and annotated according to the marker genes (Supplementary Table S2). The ‘FindMarkers’ and ‘FindAllMarkers’ functions in Seurat were used to identify DEGs using the following criteria: lnFC > 0.25, *p* < 0.05, min.pct > 0.1. Go analysis of the biological process enrichment of DEGs in each cluster or subset was performed by the Clusterprofiler package (v.3.14.3).

### Gene signatures analysis

The CXCL13, Tfh, TLS signatures were derived from a review recently published by Sautes-Fridman et al. [23]. Genes included in the Tfh signature are CXCL13, CD200, FBLN7, ICOS, SGPP2, SH2D1A, TIGIT, and PDCD1. Genes included in the 12-chemokine signature for detection of TLS are CCL2, CCL3, CCL4, CCL5, CCL8, CCL18, CCL19, CCL21, CXCL9, CXCL10, CXCL11, and CXCL13. According to the therapeutic response, the samples were divided into naive, non-MPR, and MPR groups. GSEA analysis was performed on the three signature gene sets in different therapeutic groups, and heat maps were drawn according to the enrichment score results. *P*adj > 0.05 in the enrichment pathway was adjusted to 0 to draw the heat map.

### Shared clonotypes between tissues

TCR clone data were collected according to CD4 and CD8 clusters, respectively. We obtained 40,329 CD4^+^ T cells and 50,884 CD8^+^ T cells, of which 75.09% of CD4^+^ T cells and 68.64% of CD8^+^ T cells had TCR sequenced, respectively. Shared TCR clonotypes between tissues were defined as TCRs with the same complementarity-determining region 3 (CDR3) nucleotide sequences. The TCR clone data were divided into P0, P1, D, N, T, and LN groups according to the sample tissue type, and we used the R package VennDiagram to draw the Venn diagram and see the overlap between different tissue types.

### Diversity and clonality of CD4^+^ and CD8^+^ T cells

The diversity of the TCR repertoire was calculated based on the Shannon–Wiener index (Shannon index), which is a function of both the relative number of clonotypes present and the relative abundance or distribution of each clonotype. The Shannon index is calculated as follows.

$${{{\mathrm{Shannon}}}}\;{{{\mathrm{index}}}} = - \mathop {\sum}\nolimits_i {\frac{{n_i}}{N}lg\frac{{n_i}}{N}}$$

In the Shannon index, *n*_*i*_ is the clonal size of the ith clonotype (i.e., the number of copies of a specific clonotype), and *N* is the total number of TCR receptor sequences analyzed.

The clonality is calculated according to the formula:

$${{{\mathrm{Clonality}}}} = 1 - \frac{{ - \mathop {\sum }\nolimits_{i = 1}^N p_i {\log}_2(p_i)}}{{{\log}_2(N)}}$$

where *p*_*i*_ is the proportional abundance of the rearrangement *i*, and *N* is the total number of TCR receptor sequences analyzed.

### STARTRAC analysis

STARTRAC analysis of clonal expansion and transition was done as described in [64]. Code for STARTRAC analysis is available at https://github.com/Japrin/STARTRAC. Scores and significance for CD8^+^ T clusters (C3, C5, C7, C8, C21, and C24) and CD4^+^ T clusters (C2, C4, and C11) were calculated separately. The p values were determined by the Wilcoxon test.

### Putative interactions between cell clusters

CellPhoneDB (v.2.0) was used to perform a systematic analysis of cell–cell communications [65]. It scores L-R pairs based on p-values of the mean score. Briefly, we looked for the cell-type specific L-R interactions to identify the most relevant interactions between cell types. Only receptors and ligands expressed in more than 10% of the cells in the specific cluster were considered significant. We did pairwise comparisons for all cell types. First, we randomly arranged the cluster genes of all cells 1000 times to determine the mean receptor expression level of a cluster and the mean ligand expression level of the interacting cluster. For multimeric receptors and/or ligands, we required that all subunits of the complex are expressed, and we used the minimum average expression member of the complex to perform the random shuffling. This produces a null distribution for each ligand-receptor pair in each pairwise comparison between two clusters. By calculating the proportion of means that were equal to or above the actual mean, we obtained a p-value for the likelihood of cell-type specificity for a given receptor-ligand complex. We then prioritized the interactions based on the number of important L-R pairings between clusters, and manually selected the biologically relevant ones.

### Multiplex immunohistochemistry staining and TLS quantification

Multiplex fluorescent staining was obtained using Opal 7-Color Manual IHC Kit (PerkinElmer, Cat# NEL811001KT) according to the manufacturer’s instruction. The primary antibodies and IHC metrics were: rabbit monoclonal anti-human CD4 antibody (Abcam, Cat# ab133616, diluted at 1:1000), rabbit monoclonal anti-human CD20 antibody (Abcam, Cat# ab78237, diluted at 1:2000), mouse monoclonal anti-human IgG antibody (Abcam, Cat# ab200699, diluted at 1:800), rabbit polyclonal anti-human IL-21 antibody (Invitrogen, Cat# PA5-34801, diluted at 1:2000), mouse monoclonal anti-human CD21 antibody (Invitrogen, Cat# MA5-11417, diluted at 1:200), mouse monoclonal anti-human FoxP3 antibody (Abcam, Cat# ab20034, diluted at 1:500), rabbit monoclonal anti-human IgG1 antibody (Abcam, Cat# ab108969, diluted at 1:1000), rabbit monoclonal anti-human IgG3 antibody (Abcam, Cat# ab193172, diluted at 1:800), recombinant rabbit monoclonal anti-human IgA antibody (Invitrogen, Cat# MA5-32575, diluted at 1:800). The slides were microwave heat-treated after each tyramide signal amplification operation. Nuclei were stained with DAPI after all the antigens above had been labeled. To obtain multispectral images, the stained slides were scanned using the Mantra System (PerkinElmer) following the manufacturer’s instructions to generate.im3 image cubes for downstream analysis. To analyze the spectra for all fluorophores included, inForm image analysis software (v2.4.4; PerkinElmer) was used. For each slide, 10 fields of immune cell-enriched area were selected for image capture.

For TLS quantification, the stained slides were panoramic scanned and visualized using the TissueFAXSi­plus imaging system (TissueGnostics, Vienna, Austria; acquisition software: TissueFAXS v7.0.6245) equipped with a digital Pixelink color camera (PCO AG). Multispectral images were analyzed with StrataQuest software v7.0.1.165 (TissueGnostics). Structures were identified as aggregates of lymphocytes having histological features with analogous structures to that of lymphoid tissue with germinal centers (including B cells (CD20), T cells (CD4/CD8), and follicular dendritic cells (CD21), appearing in the tumor area. TLS density is defined as the total number of structures identified either within the tumoral area or in direct contact with the tumoral cells on the margin of the tumors (numbers of TLS per mm^2^ area).

### Plasma IL-21 ELISA

Plasma IL-21 levels were detected by Human IL-21 Uncoated ELISA kits with a standard curve range of 8–1000 pg/ml (Invitrogen, Cat# 88-8218-22) following the manufacturer’s instructions. Optical densities were measured at 450 nm.

### Flow cytometry

Human PBMC and single-cell suspensions prepared from fresh tumors and matched tissues were used for flow cytometry analysis. One million cells were stained with Brilliant Violet 510 anti-human CD45 (Biolegend, Cat# 304035), PE anti-human LAMP3 (BD Biosciences, Cat# 556020), PE-Dazzle^TM^ 594 anti-human CD11c (Biolegend, Cat# 301641) at room temperature for 20 min. Another 1–2 million cells were co-culture with Leukocyte Activation Cocktail, with BD GolgiPlug™(BD Biosciences, Cat# 550583) in a 37 °C humidified CO_2_ incubator for 4–6 h according to the manual. Following activation, cells were harvested and washed with FACS Buffer. Then cell surface staining was performed in FACS buffer containing antibody cocktails (Brilliant Violet 510 anti-human CD45, Biolegend, Cat# 304035; FITC anti-human CD4, Biolegend, Cat# 317408; PE-Cy7 anti-human CD8, BD Biosciences, Cat# 557654; PE anti-human CD25, Biolegend, Cat# 302606; PerCP/Cy5.5 anti-human CD127(IL-7Rα), Biolegend, Cat# 351322; APC-Cy7 anti-human CD19, Biolegend, Cat# 302217) at room temperature for 20 min. After washing twice with FACS buffer, the cells were fixed using a FoxP3/Transcription Staining Buffer Set (eBioscience™ Foxp3/Transcription Factor Staining Buffer Set, Cat# 00-5523) according to the manufacturer’s instructions. Cells were washed twice with 1× permeabilization buffer (eBioscience, Cat# 00-8333), and then intracellularly stained with FoxP3 (Alexa Fluor 647 anti-human FOXP3, Biolegend, Cat# 320114) and IL-21 (Brilliant Violet 421 anti-human IL-21, BD Biosciences, Cat# 564755).

Cells were washed twice with wash buffer and then analyzed on the BD-Aria II flow cytometer (BD Biosciences, San Diego, CA, USA) and analyzed using FlowJo software (v.10.1, FlowJo LLC, Ashland, OR, USA).

### Statistical analysis

The data were analyzed using GraphPad Prism (v.8.0.1, GraphPad Software, La Jolla, California, USA). Data are presented as the mean ± standard error of the mean (SEM). Comparisons of numerical variables among more than two groups were assessed by ordinary one-way ANOVA or the Kruskal–Wallis test. Comparisons of paired numerical variables between two groups were assessed by Wilcoxon matched-pairs signed-rank test, and unpaired differences between two groups were assessed by the Mann–Whitney test. A *p* value < 0.05 was considered significant (*p < 0.05, **p < 0.01, ***p < 0.001, ***p < 0.001, ns: not significant).

## Supplementary information

Supplementary Figure S1

Supplementary Figure S2

Supplementary Figure S3

Supplementary Figure S4

Supplementary Figure S5

Supplementary Figure S6.

Supplementary Figure S7

Supplementary Table S1

Supplementary Table S2

Supplementary Table S3

Supplementary Table S4

Supplementary File S1

aj-checklist

CDD-author contribution form

## Data Availability

The datasets used during the current study are available from the corresponding author on reasonable request as another research is being conducted.

## Code availability

This study did not generate any unique code. All code or software tools used in this study follow the official tutorial and are freely or commercially available.
