# Supplementary figures and images for: Single-cell profiling of immune cells after neoadjuvant pembrolizumab and chemotherapy in IIIA non-small cell lung cancer (NSCLC)
Source: Cell Death Dis. 2022 Jul 13;13(7):607. doi: 10.1038/s41419-022-05057-4 (PMC9279493; doi:10.1038/s41419-022-05057-4)

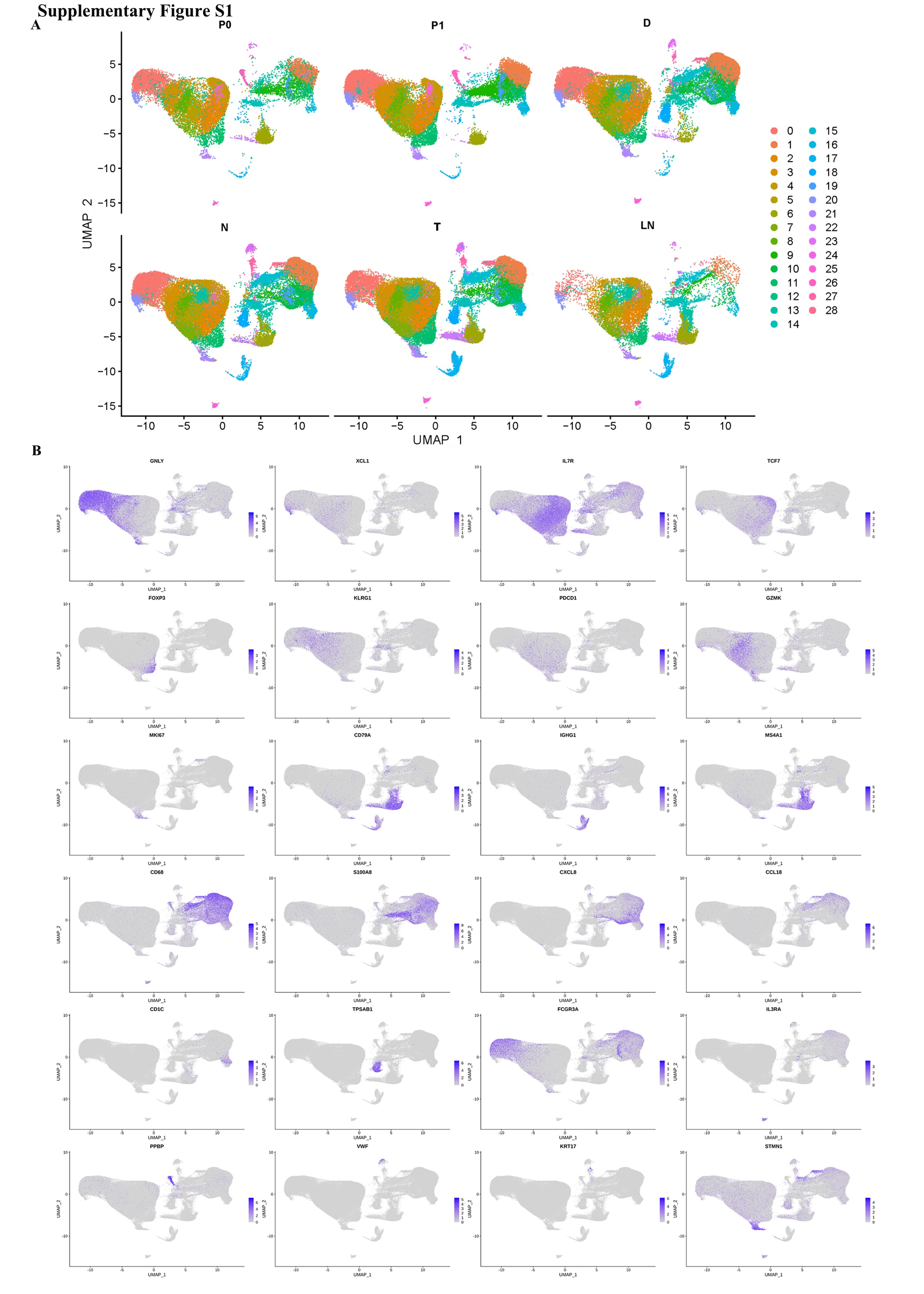

Supplement: Supplementary file 1 — Supplementary Figure S1 [file 41419_2022_5057_MOESM1_ESM.tif]

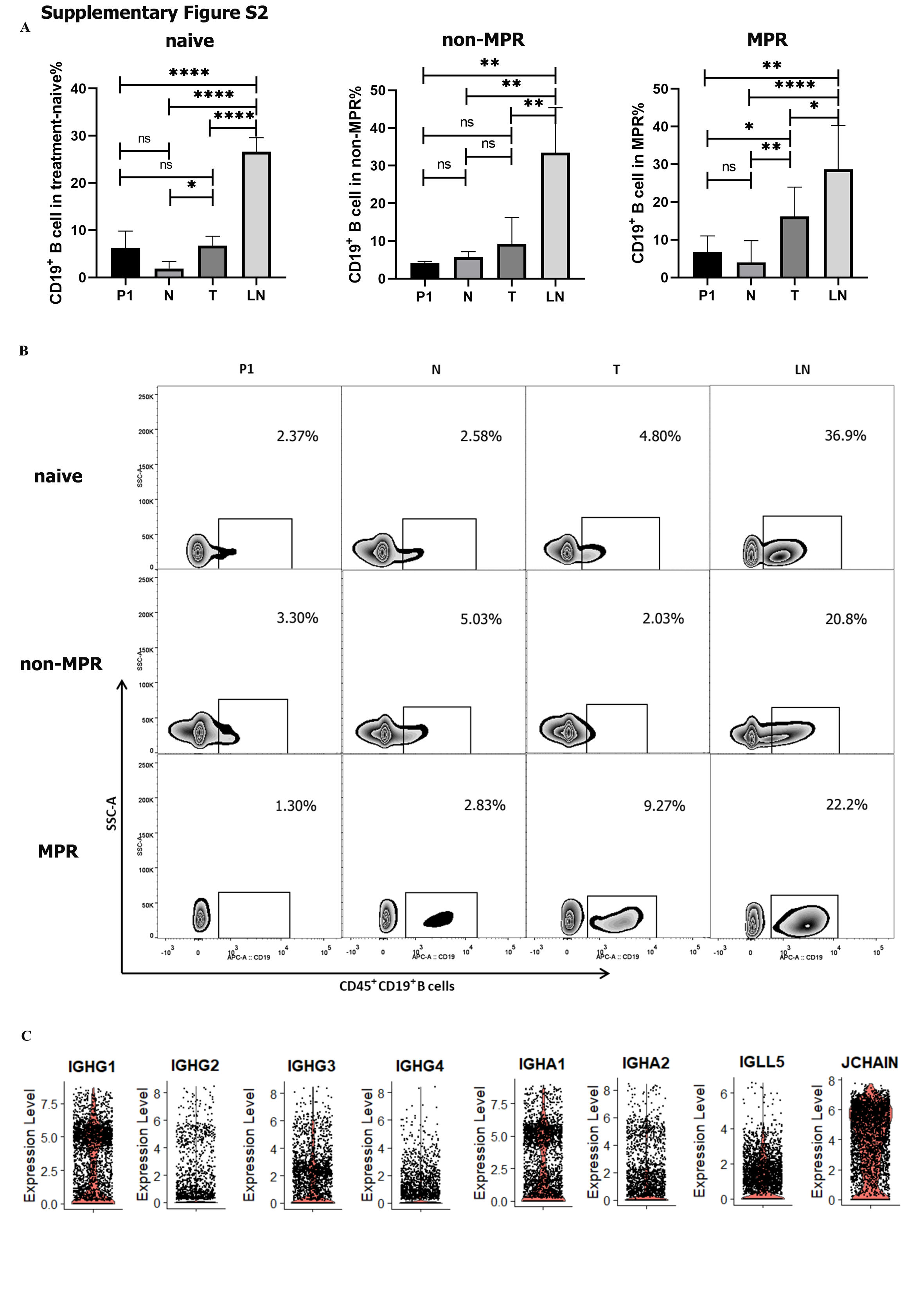

Supplement: Supplementary file 2 — Supplementary Figure S2 [file 41419_2022_5057_MOESM2_ESM.tif]

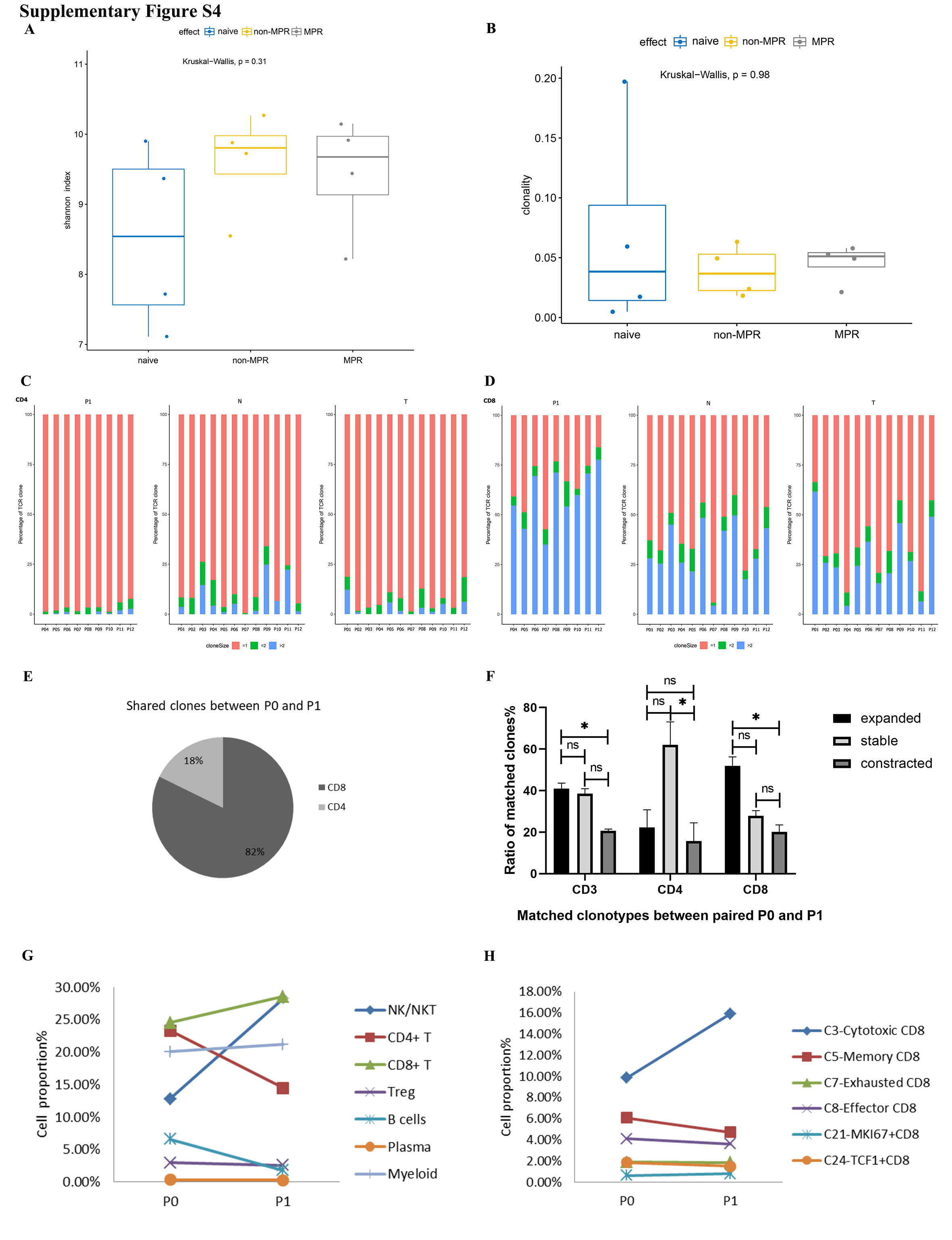

Supplement: Supplementary file 4 — Supplementary Figure S4 [file 41419_2022_5057_MOESM4_ESM.tif]

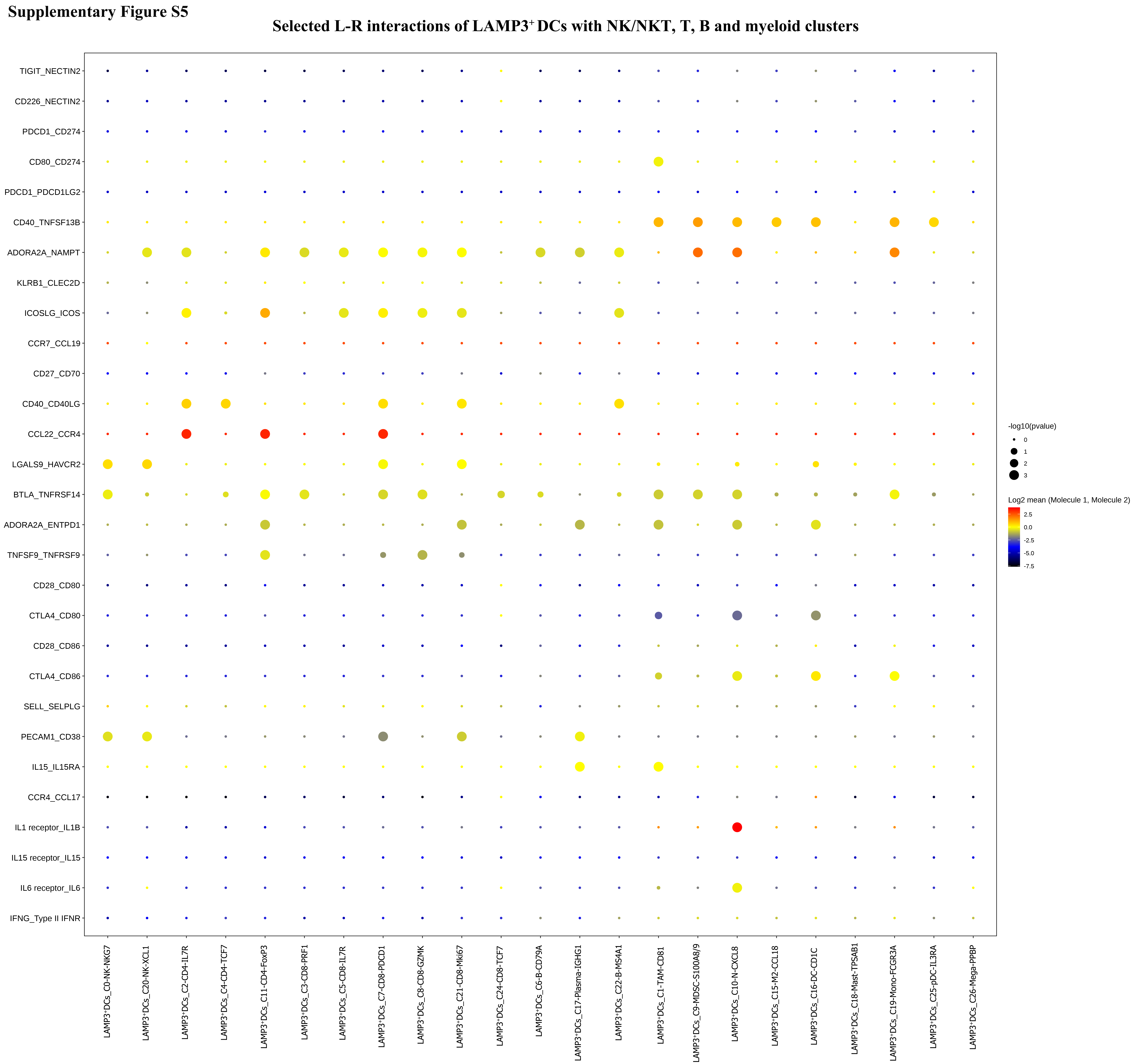

Supplement: Supplementary file 5 — Supplementary Figure S5 [file 41419_2022_5057_MOESM5_ESM.tif]

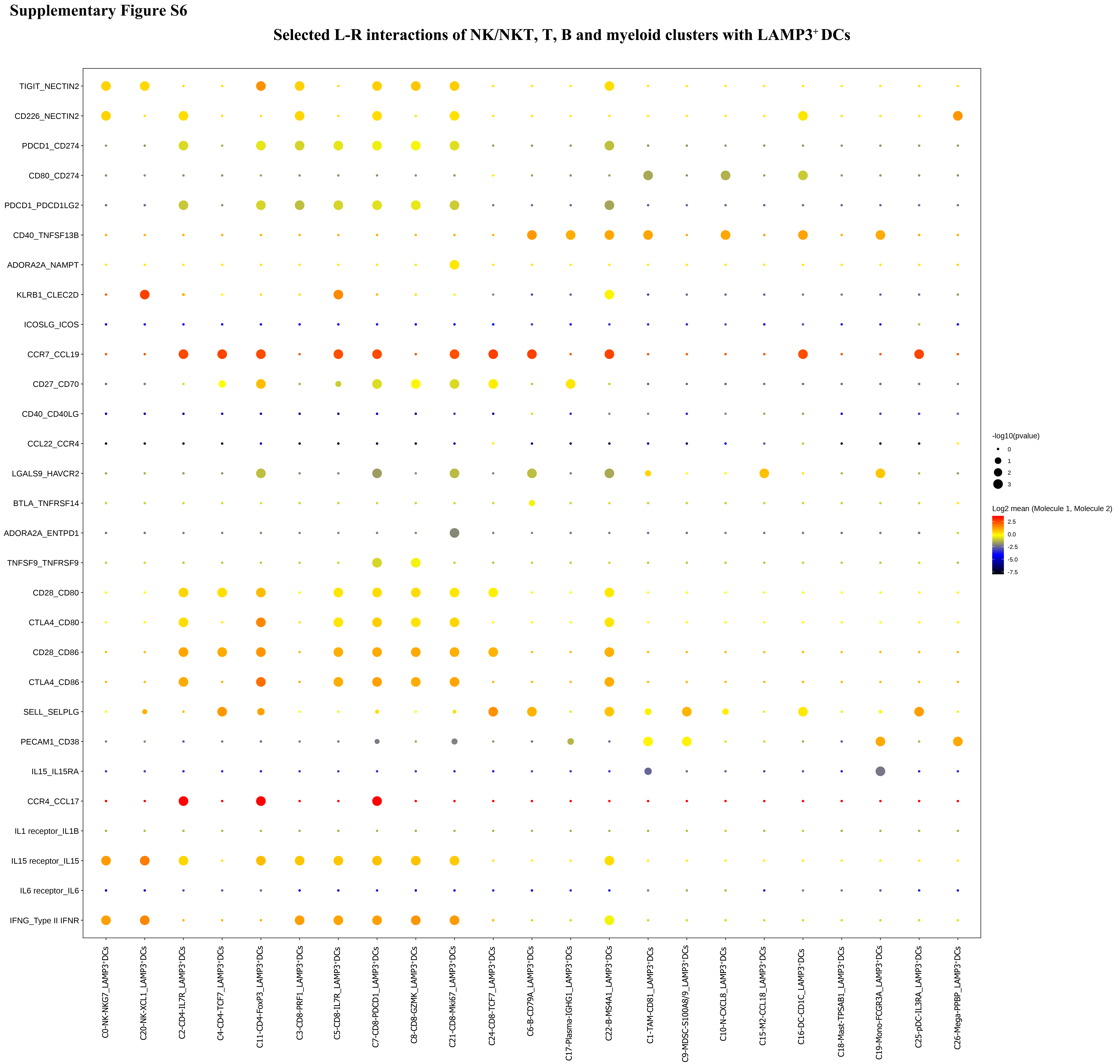

Supplement: Supplementary file 6 — Supplementary Figure S6. [file 41419_2022_5057_MOESM6_ESM.tif]

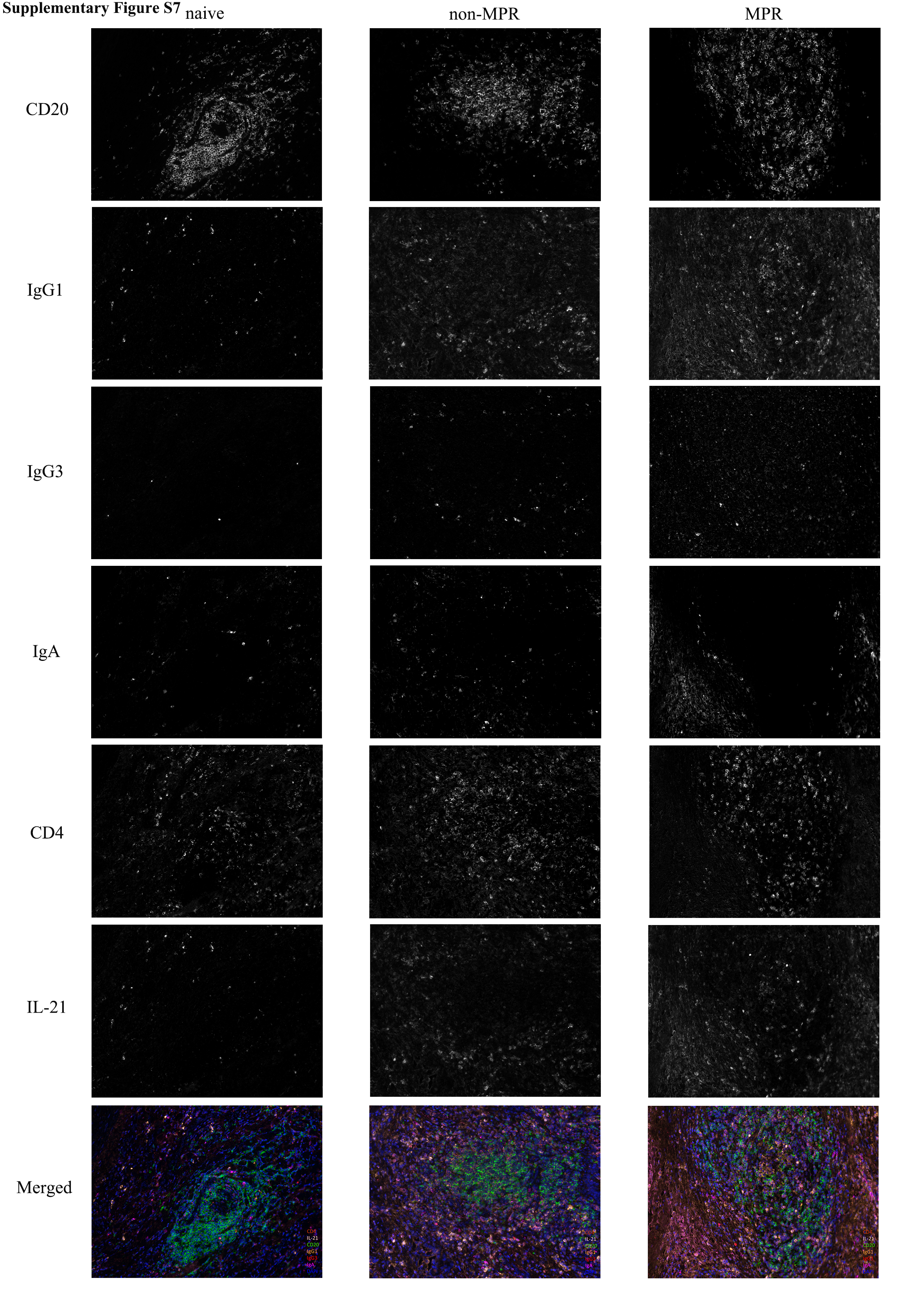

Supplement: Supplementary file 7 — Supplementary Figure S7 [file 41419_2022_5057_MOESM7_ESM.tif]
