# Supplementary material for: Single-cell profiling of immune cells after neoadjuvant pembrolizumab and chemotherapy in IIIA non-small cell lung cancer (NSCLC)
Source: Cell Death Dis. 2022 Jul 13;13(7):607. doi: 10.1038/s41419-022-05057-4 (PMC9279493; doi:10.1038/s41419-022-05057-4)
Supplement: Supplementary file 8 — Supplementary Table S1 [file 41419_2022_5057_MOESM8_ESM.docx]

| Preoperative treatment | Patient | Age | Sex | Smoking  history | PD-L1  (TPS) | Pathology | RVT% | Pathologic response |
| --- | --- | --- | --- | --- | --- | --- | --- | --- |
| Naive | P01 | 75 | Male | Yes | N/A | AD | N/A | N/A |
|  | P02 | 65 | Female | No | N/A | AD | N/A | N/A |
|  | P03 | 73 | Male | Yes | N/A | SCC | N/A | N/A |
|  | P04 | 58 | Male | No | N/A | AD | N/A | N/A |
| Neoadjuvant | P05 | 59 | Male | Yes | 50% | SCC | 10% | MPR |
|  | P06 | 69 | Male | Yes | 5% | SCC | 95% | non-MPR |
|  | P07 | 51 | Female | Yes | 0% | SCC | 0% | MPR |
|  | P08 | 66 | Female | No | 90% | AD | 80% | non-MPR |
|  | P09 | 67 | Male | No | 3% | SCC | 0% | MPR |
|  | P10 | 62 | Male | Yes | 30% | SCC | 0% | MPR |
|  | P11 | 46 | Male | No | 60% | AD | 60% | non-MPR |
|  | P12 | 68 | Male | Yes | 15% | SCC | 95% | non-MPR |

**Supplementary Table S1. The basic clinicopathological characteristics of patients in single-cell sequencing cohort.**

PD-L1, programmed cell death-ligand 1; TPS, tumor proportion score; AD, adenocarcinoma; SCC, squamous cell carcinoma; RVT, residual viable tumor cells; N/A, not applicable; MPR, major pathologic response.
