# Supplementary material for: Single-cell profiling of immune cells after neoadjuvant pembrolizumab and chemotherapy in IIIA non-small cell lung cancer (NSCLC)
Source: Cell Death Dis. 2022 Jul 13;13(7):607. doi: 10.1038/s41419-022-05057-4 (PMC9279493; doi:10.1038/s41419-022-05057-4)
Supplement: Supplementary file 9 — Supplementary Table S2 [file 41419_2022_5057_MOESM9_ESM.docx]

**Supplementary Table S2. Cell cluster identification and annotation.**

We identified and annotated cell types based on the markers of each cluster. Cell markers were used as follows:

| Cell types | Cell cluster | Marker genes |
| --- | --- | --- |
| NK/NKT | C0-NK/NKT-GNLY | GNLY, FGFBP2, NKG7 |
|  | C20-NK-XCL1 | XCL1, XCL2, KLRB1, GNLY, NKG7 |
| CD4^+^ T cells | C2-CD4-IL7R | LTB, IL7R, MAL, CD3E, CD3D, CD4 |
|  | C4-CD4-TCF7 | TCF7, LTB, LEF1, CD3E, CD3D, CD4 |
|  | C11-Treg-FoxP3 | TNFRSF4, BATF, FOXP3, CTLA4, CD4 |
| CD8^+^ T cells | C3-CD8-KLRG1 | CD8A, CD8B, GNLY, NKG7, KLRG1, PRF1 |
|  | C5-CD8-IL7R | IL7R, CD8A, CD8B, KLRB1, CD3E |
|  | C7-CD8-PDCD1 | DUSP4, CD8A, CD8B, CD3D, CD3E, PDCD1, CTLA4, LAG3 |
|  | C8-CD8-GZMK | GZMK, CD8A, CD8B, CCL5, CD3E, CD3D |
|  | C21-CD8-MKI67 | STMN1, HIST1H4C, HMGN2, MKI67, CD8A, CD8B |
|  | C24-CD8-TCF7 | CD8B, LEF1, TCF7, SELL, CCR7, CD8A, CD8B, CD3D, CD3E |
| B and plasma cells | C6-B cells-CD79A | CD79A, CD79B, MS4A1, BANK1, CD19 |
|  | C17-Plasma cells-IGHG1 | IGK、IGL、IGH family genes, MZB1, CD79A |
|  | C22-B cells-MS4A1 | CD79A, CD79B, MS4A1, BANK1, CD19 |
| Myeloid cells | C1-TAM-CD81 | CD68, APOC1, FABP4, C1QB, APOE, CD81 |
|  | C9-MDSC-like macrophage-S100A8/A9 | S100A8, S100A12, S100A9, VCAN, FCN1 |
|  | C10-Neotrophil-CXCL8 | CXCL18, IL1B, THBS1 |
|  | C15-Macrophage-CCL18 | APOC1, CCL18, APOE, C1QB, FABP4, CD68 |
|  | C16-DC-CD1C | CD1C, HLA II molecules, CD74, CD83, CD86 |
|  | C18-Mast cells-TPSAB1 | TPSAB1, TPSB2, MS4A2 |
|  | C19- Monocyte-FCGR3A | COTL1, FCN1, FCGR3A, CD68 |
|  | C25-pDC-IL3RA | IL3RA, GZMB, LILRA4 |
|  | C26-Megakaryocyte-PPBP | PPBP, PF4 |
| Cycling cells | C14-MALAT1 | MALAT1, NEAT1, PTPRC |
|  | C28-STMN1 | STMN1, TUBA1B, HMGN2, TYMS |
| Others | C23-Endothelial cells-VWF | VWF, CAV1, PECAM1, GNG11 |
|  | C27-Epithelial cells-KRT17 | KRT family genes, EPCAM |
