# Supplementary material for: Single-cell profiling of immune cells after neoadjuvant pembrolizumab and chemotherapy in IIIA non-small cell lung cancer (NSCLC)
Source: Cell Death Dis. 2022 Jul 13;13(7):607. doi: 10.1038/s41419-022-05057-4 (PMC9279493; doi:10.1038/s41419-022-05057-4)
Supplement: Supplementary file 10 — Supplementary Table S3 [file 41419_2022_5057_MOESM10_ESM.docx]

**Supplementary Table S3. Clinicopathological characteristics of included NSCLC patients in single-cell sequencing and validation cohorts.**

| Characteristics | Single-cell (N=12) | Validation (N=56) | p value |
| --- | --- | --- | --- |
| Age (years) |  |  | 0.528 |
| ≤60  ＞60 | 4 (33.3%)  8 (66.7%) | 26 (46.4%)  30 (53.6%) |  |
| Sex |  |  | 1.000 |
| Male | 9 (75.0%) | 44 (78.6%) |  |
| Female | 3 (25.0%) | 12 (21.4%) |  |
| Smoking history |  |  | 0.738 |
| No | 5 (41.7%) | 18 (32.1%) |  |
| Yes | 7 (58.3%) | 38 (67.9%) |  |
| cTNM |  |  | 0.631 |
| IIIA | 12 (100%) | 53 (94.6%) |  |
| IIIB | 0 (0%) | 3 (5.4%) |  |
| Pathology |  |  | 1.000 |
| adenocarcinoma | 5 (41.7%) | 17 (30.4%) |  |
| squamous | 7 (58.3%) | 36 (64.3%) |  |
| other | 0 (0%) | 3 (5.3%) |  |
| Neoadjuvant |  |  | 0.528 |
| No | 4 (33.3%) | 26 (46.4%) |  |
| Yes | 8 (66.7%) | 30 (53.6%) |  |
| Tumor location |  |  | 1.000 |
| Left | 5 (41.7%) | 24 (42.9%) |  |
| Right | 7 (58.3%) | 32 (57.1%) |  |
| Type of resection |  |  | 0.884 |
| Pneumonectomy | 1 (8.3%) | 6 (10.7%) |  |
| Lobectomy | 7 (58.4%) | 37 (66.1%) |  |
| Sleeve lobectomy | 4 (33.3%) | 13 (23.2%) |  |
