# Supplementary material for: Single-cell profiling of immune cells after neoadjuvant pembrolizumab and chemotherapy in IIIA non-small cell lung cancer (NSCLC)
Source: Cell Death Dis. 2022 Jul 13;13(7):607. doi: 10.1038/s41419-022-05057-4 (PMC9279493; doi:10.1038/s41419-022-05057-4)
Supplement: Supplementary file 11 — Supplementary Table S4 [file 41419_2022_5057_MOESM11_ESM.docx]

**Supplementary Table S4. Comparison of mIHC staining markers in different treatment response groups in patients with adenocarcinoma and squamous cell carcinoma.**

| Markers | Adenocarcinoma | | | p value | Squamous cell carcinoma | | | p value |
| --- | --- | --- | --- | --- | --- | --- | --- | --- |
|  | naïve  (n=9) | non-MPR  (n=5) | MPR  (n=3) |  | naïve  (n=17) | non-MPR  (n=5) | MPR  (n=14) |  |
| CD20 | 11.04% | 11.82% | 14.39% | 0.7809 | 9.87% | 9.00% | 15.76% | 0.0121 |
| CD4 | 5.63% | 6.36% | 7.47% | 0.2576 | 6.25% | 3.94% | 8.99% | 0.0018 |
| IL-21 | 0.79% | 1.51% | 2.08% | 0.3032 | 1.58% | 0.73% | 2.01% | 0.0165 |
| IgG1 | 0.84% | 1.61% | 2.30% | 0.1063 | 1.65% | 1.51% | 2.94% | 0.0105 |
| IgG3 | 0.22% | 0.25% | 0.36% | 0.2151 | 0.36% | 0.28% | 0.61% | 0.0014 |
| IgA | 1.68% | 1.16% | 0.33% | 0.0512 | 1.86% | 1.56% | 0.57% | 0.0001 |

p value was determined by Kruskal-Wallis test.
